# Synthesis of a Tetrahedral Metal–Organic Supramolecular Cage with Dendritic Carbazole Arms

**DOI:** 10.3390/ijms232415580

**Published:** 2022-12-08

**Authors:** Juanzi Lianglu, Weinan Hu, Xinju Zhu, Hong-Yu Zhang, Linlin Shi, Xin-Qi Hao, Mao-Ping Song

**Affiliations:** 1 College of Chemistry, Zhengzhou University, Zhengzhou 450001, China; 2 School of Basic Medical Science, Zhengzhou University, Zhengzhou 450001, China

**Keywords:** metal–organic supramolecular cage, self-assembly, aggregation-induced emission

## Abstract

In recent years, incredible endeavors have been devoted to the design and self-assembly of discrete metal–organic cages (MOCs) with expanding intricacy and functionality. The controlled synthesis of metal–organic supramolecular cages with large branched chains remains an interesting and challenging work in supramolecular chemistry. Herein, a tetrahedral metal–organic supramolecular cage (Zn^II^_4_L_4_) containing 12 dendritic carbazole arms is unprecedentedly constructed through coordination-driven subcomponent self-assembly and characterized in different ways. Interestingly, tetrahedral supramolecular **Cage-1** exhibited the potential for aggregation-induced emission (AIE) performance and stimulus-responsive luminescence features, and it achieved color-tunable photoluminescence due to the introduction of dendritic carbazole arms. Crucially, owing to the great photophysical properties of **Cage-1** in solution, **Cage-1** was enabled to act as a fluorescent ink for the vapor-responsive recording and wiping of information.

## 1. Introduction

Supramolecular chemistry is an emergent subject that is widely known and extensively studied on account of its unique properties and potential applications in biological imitation [1,2,3], gas encapsulation [4], organic photoreactions [5,6], catalysis [7,8], molecular recognition [9,10,11], and so on. Coordination-driven self-assembly [12,13,14,15,16,17,18,19,20,21] is a simple and efficient way of constructing three-dimensional (3D) supramolecular complexes, such as metal–organic nanocages [22,23], metal–organic frameworks [24,25], metallacycles [20,26,27], etc. A multitude of metal–organic supramolecular cages have been handily constructed in a variety of shapes, such as octahedrons [28], tetrahedrons [29], spheres [30], square prisms [31], triangular prisms [32], spirals [33], capsules [34], etc. In addition, breakthrough research has been conducted in the design strategy of metal-ion coordination-induced supramolecular materials over the last two years [35,36]. Nevertheless, complex supramolecular cages with large branched chains remain highly underexplored.

Incorporating an aggregation-induced emission (AIE) fluorophore [37,38] and combining piezochromic or vapochromic chromophores [39,40,41] are common strategies for the preparation of advanced optical materials. As is well-known, the phenomenon of AIE was initially discovered by Tang’s group in 2001, and they subsequently proposed the corresponding luminescence mechanism: that the restriction of intramolecular rotation (RIR) caused the energy of the excited state to be released as a form of radiation, such as fluorescence or phosphorescence [42]. Based on this, AIE theories have been used to design advanced optical materials. In particular, gas-sensitive optical materials based on AIE show unique advantages in the fields of anticounterfeiting and data encryption [43,44,45].

Dendrimers are monodisperse, highly branched macromolecules that are constructed from an interior core with a regular array of branching units. Carbazole is attractive as a typical dendrimer that has strong potential for luminescence and a dendritic structure [46,47,48]. Therefore, novel dendrimers based on carbazole as a modification unit, with a tetrahedral metal–organic supramolecular cage (Zn^II^_4_L_4_) formed by coordinated self-assembly as the core, may exhibit fascinating photophysical capabilities. To our knowledge, the modification of tetrahedral supramolecular cages with novel dendrimers based on carbazole is very rare. Herein, the synthesis of ligand **LA** with a dendritic carbazole arm and the obtained tetrahedral supramolecular **Cage-1** (Zn^II^_4_L_4_) with 12 dendritic carbazole arms (containing 36 carbazole molecules) via coordinated self-assembly is presented. Its photophysical properties have also been researched.

## 2. Results and Discussion

**Scheme 1:**
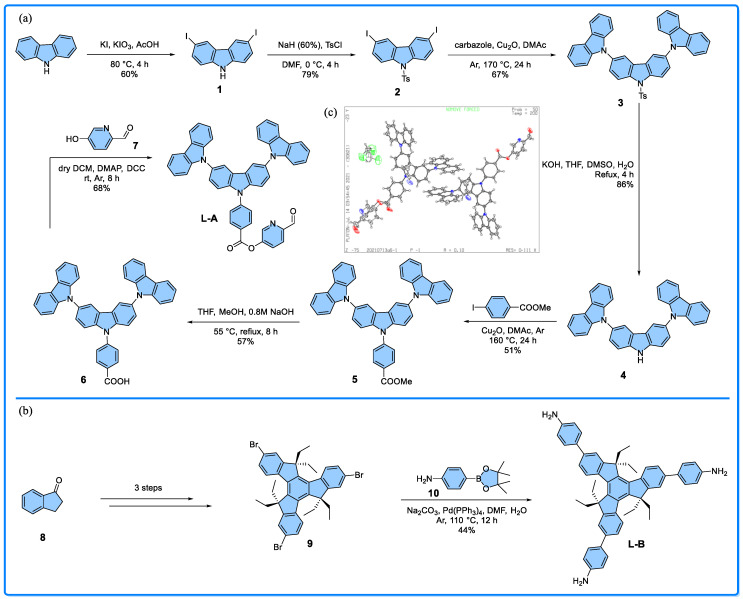
(**a**) The synthesis of ligand **LA**; (**b**) The synthesis of ligand **LB**; (**c**) The crystal structure of ligand **LA**.

Both ligand **LA** and ligand **LB** were synthesized with excellent yields (Scheme 1a,b). Ligand **LA** was synthesized in seven steps. Commercially available carbazole was selected as the starting substrate for the synthetic route. Under acidic conditions, iodine was formed by comproportionation with potassium iodide and potassium iodate, which then reacted with the carbazole to obtain Compound **1** for a 60% yield. Intermediate **2** was obtained by converting the amidogen in Compound **1** to the p-toluene sulfonyl group by treatment with NaH (60%) and 4-Methoxybenzenesulfonyl chloride (TsCl) for a 79% yield. Under inert gas protection, Compound **3** was obtained from carbazole and Compound **2**, catalyzed by Cu_2_O in *N,N*-dimethylacetamide (DMAc) at 170 °C for 24 h, via the Ullmann condensation reaction for a yield of 67%. Subsequently, the N-Ts bond in Compound **3** was cleaved in an alkaline environment to obtain Compound **4** for an 86% yield. Compound **5** was synthesized via the Ullmann coupling reaction using Compound **4** and 4-iodo-benzoic acid methyl ester as ingredients for a 51% yield. Compound **5** was hydrolyzed under alkaline conditions to obtain Compound **6** for a 57% yield. Then, the key ligand, **LA**, was synthesized from Compound **6** and Compound **7**, catalyzed by 4-dimethyl-aminopyridine (DMAP) in dichloromethane (DCM) at room temperature for 8 h, via the ester condensation reaction for a yield of 68%. Notably, the slow volatility of the solution of acetonitrile and chloroform (v:v = 1:1), which contained ligand **LA**, at room temperature provided crystals suitable for X-ray crystallographic analysis (Scheme 1c, Figure S17). Finally, ligand **LB** was prepared from Compound **9** and Compound **10** through a Suzuki coupling reaction under the catalysis of tetra-triphenylphosphine palladium for a yield of 44%. The chemical structures of all the synthesized compounds were determined by NMR and ESI-MS (Figures S1–S12).

**Scheme 2:**
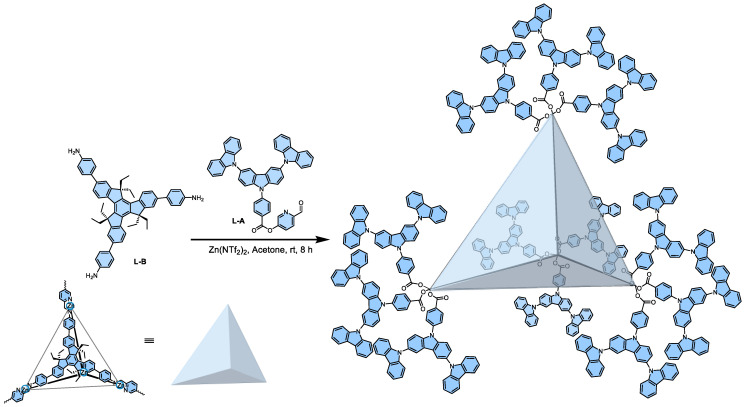
The synthesis of **Cage-1**.

Owing to the successful characterization of ligands **LA** and **LB**, the tetrahedral supramolecular **Cage-1** (Zn^II^_4_L_4_) with 12 dendritic carbazole arms was designed and synthesized using Zn(NTf_2_)_2_ as the coordination metal (Scheme 2, Figure S13). In general, the coordinated self-assembly of the metal–organic supramolecular cages was carried out in acetonitrile solution [49]. Thus, **LA**, **LB**, and Zn(NTf_2_)_2_ self-assembled in acetonitrile solvent were applied in an initial attempt to form a supramolecular cage. We observed the experimental phenomenon, and the reaction system showed a heterogeneous state in acetonitrile. Unfortunately, the target supramolecular cage had difficulty forming under such circumstances. Since **LB** and Zn(NTf_2_)_2_ have great solubility in CH_3_CN and **LA** has excellent solubility in DCM solvent, the mixed solvent (CH_3_CN: DCM = 1:1) was used as a condition for the self-assembly to proceed. As the reaction progressed, the solution gradually changed from a homogeneous system to a heterogeneous system, which also hindered the formation of the target supramolecular cage. Ultimately, through continuous attempts, it was discovered that **LA**, **LB**, and Zn(NTf_2_)_2_ could remain homogeneous in acetone and maintain such a system until the end of the reaction. Accordingly, metal–organic supramolecular **Cage-1** was successfully obtained by coordinated self-assembly under this condition. Subsequently, the proportions of **LA**, **LB**, and Zn(NTf_2_)_2_ were retained for control, and we then concluded that **LA**: **LB**: Zn(NTf_2_)_2_ = 3.0:1:1.2 was the best proportion for the self-assembly of metal–organic supramolecular **Cage-1**.

**Figure 1:**
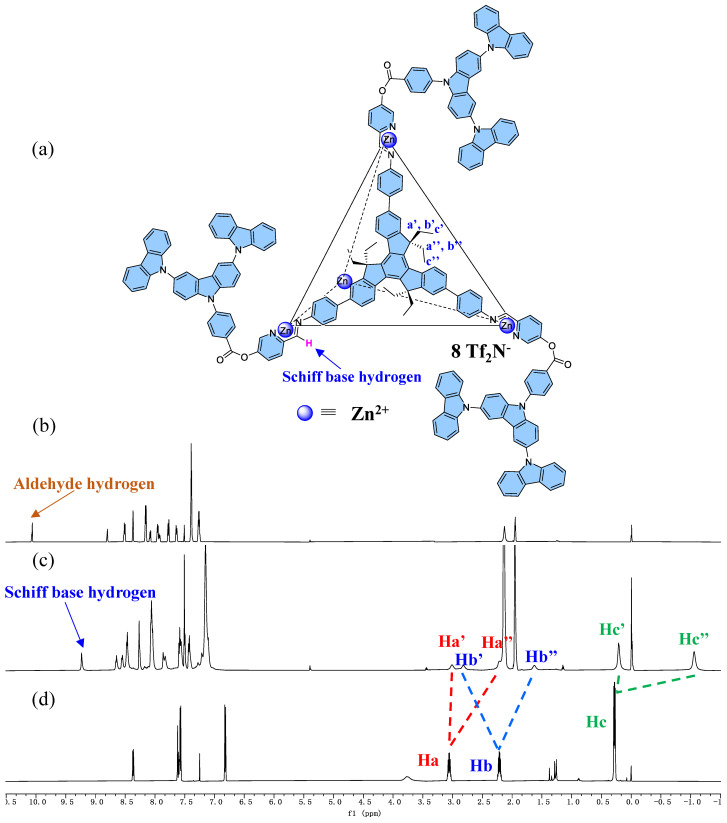
(**a**) The structure of **Cage-1**; (**b**) ^1^H NMR (CD_3_CN: CDCl_3_ = 1:1, 600 MHz, 298 K) spectrum of ligand **LA**; (**c**) ^1^H NMR (CD_3_CN: CDCl_3_ = 1:1, 600 MHz, 298 K) spectrum of **Cage-1**; (**d**) ^1^H NMR (CD_3_CN, 600 MHZ, 298 K) spectrum of ligand **LB**.

The function of Zn(NTf_2_)_2_ is indispensable in the self-assembly of tetrahedral supramolecular cages. In the absence of Zn(NTf_2_)_2_, it is extremely difficult to form dynamic imine bonds on **Cage-1**. However, in the presence of Zn(NTf_2_)_2_, the appearance of the Schiff base hydrogen signal implied the formation of dynamic imine bonds (Figure 1c). The disappearance of the hydrogen signal from the aldehyde group of ligand **LA** indicated that **LA** was completely reacted and formed the dynamic imine bonds (Figure 1b,c). After the methylene (Ha, Hb) and methyl (Hc) groups on ligand **LB** formed **Cage-1**, this brought part of the hydrogens into the cavity of **Cage-1**; thus, part of the hydrogen was shielded, moved to the high field, and then split into Ha′, Ha″, Hb′, Hb″, Hc′, and Hc″ (Figure 1c,d). The splitting of this signal proves the successful preparation of **Cage-1**. In summary, Zn(NTf_2_)_2_ not only acts as a coordination metal, but also as a catalyst in the process of **Cage-1** formation; the presence of Schiff base hydrogen and the splitting signal of the methylene (Ha, Hb) and methyl (Hc) groups on **Cage-1** support the presence of **Cage-1** as well.

To probe its stability, neat solid **Cage-1** was characterized by thermogravimetric analysis (TGA). The TGA trace of neat solid **Cage-1** (Figure S20) showed no significant loss of mass between room temperature and 324 °C, above which, decomposition was observed. In addition, powder X-ray diffraction (PXRD) of **Cage-1** revealed no distinct sharp peaks, consistent with the presence of amorphous material (Figure S19). Moreover, the stretching vibration peak of an imine double bond (1688.2 cm^−1^) can be clearly observed in the Fourier-transform infrared (FTIR) spectrum of **Cage-1** (Figure S21); moreover, the disappearance of the stretching vibration peaks of the amino group (3457.2 cm^−1^, 3371.4 cm^−1^, and 3216 cm^−1^) in the FTIR spectrum of **LB** (Figure S22) and the carbonyl stretching vibration peak of pyridine-formaldehyde (1706.3 cm^−1^) in FTIR spectrum of **LA** (Figure S23) can be observed, indicating that ligands **LA** and **LB** successfully formed an imine bond in the presence of Zn(NTf_2_)_2_, forming **Cage-1** through coordination-driven self-assembly.

**Figure 2:**
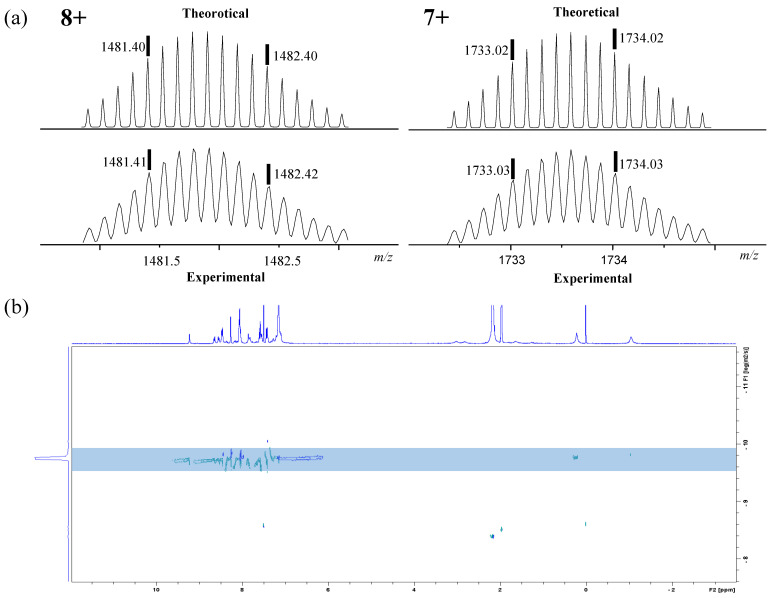
(**a**) ESI-MS spectra of **Cage-1**; (**b**) 2D DOSY spectrum of **Cage-1**.

Furthermore, in addition to nuclear magnetism resonance (NMR), electrospray ionization mass spectrometry (ESI-MS) was likewise employed to screen the formation of organic–metal supramolecular **Cage-1** (Figure 1c and Figure S14). Figure 2a shows that the ESI-MS of **Cage-1** revealed two sets of peaks with continuous charge states of 7^+^ and 8^+^, which were attributed to the successful departure of NTf_2_^−^ counterions. Experimental isotope patterns for the two peaks were highly consistent with the theoretical isotope patterns, indicating that tetrahedral metal–organic supramolecular **Cage-1** was constructed successfully via coordination-driven self-assembly. However, there is a fly in the ointment: A small amount of unattributable data was found via ESI-MS, suggesting the existence of polydispersity and multiple charge states in the dendritic carbazole arms of **Cage-1**, in contrast to those of the other cages prepared via coordinated self-assembly (Figure S13). After deconvolution, the average molar mass of self-assembled supramolecular **Cage-1** was 14087.4 Da, which is a positive match with the expected chemical composition [C_832_H_564_N_68_O_56_F_48_S_16_Zn_4_], manifesting that no other undesirable complexes or isomers existed. The 2D DOSY spectrum provides powerful supporting data for detecting whether a compound is a single structure. Therefore, the ordinate signal in the 2D DOSY spectrum indicated that there was a single compound in the system, rather than a mixture of multiple compounds (Figure 2b). Furthermore, the diffusion coefficient (D = 1.78 × 10^−10^ m^2^/s) of **Cage-1** was obtained via the DOSY spectrum, and then the estimated dynamic radius (r = 26.5 Å) of **Cage-1** could be calculated using the Stokes–Einstein equation (Figure S24). In short, ESI-MS and 2D DOSY further proved that the existence of **Cage-1** was real and singular.

**Figure 3:**
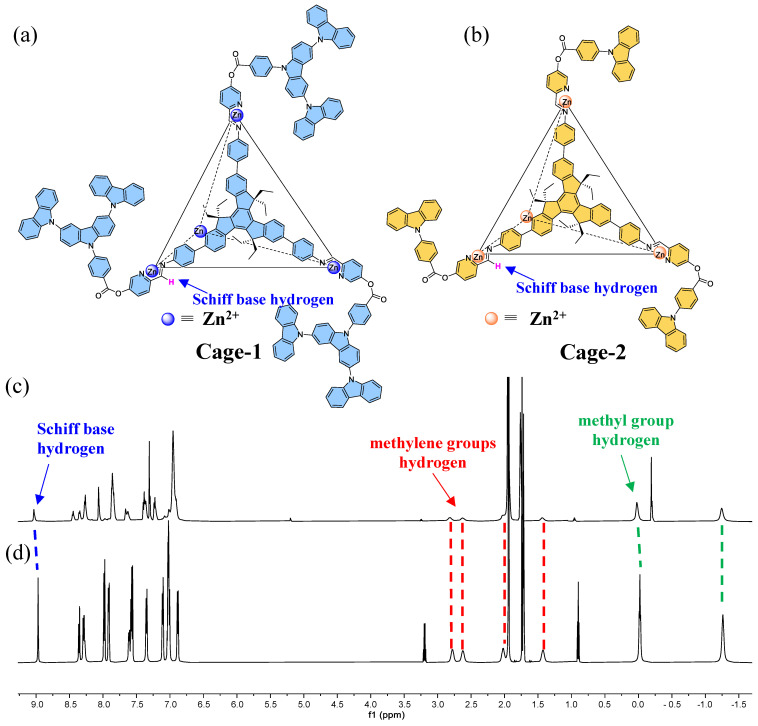
(**a**) The structure of **Cage-1**; (**b**) The structure of **Cage-2**; (**c**) ^1^H NMR (CD_3_CN: CDCl_3_ = 1:1, 600 MHz, 298 K) spectrum of **Cage-1**; (**d**) ^1^H NMR (CD_3_CN, 600 MHz, 298 K) spectrum of **Cage-2**.

We compared the ^1^H NMR of metal–organic supramolecular **Cage-2** [50], which had the same tetrahedral structure (Figure 3b), and it was preferable for supporting the successful synthesis of **Cage-1**. As shown in Figure 3c,d, the several characteristic signal peaks (Schiff base hydrogen, methylene hydrogen, and methyl hydrogen) were not much different from metal–organic **Cage-2**, only moving about 0.2–0.5 ppm to the low field, which might have been brought about by the distinction of the deuterium reagent. Therefore, the comparison with the ^1^H NMR of **Cage-2** also suggests the formation of **Cage-1**.

**Figure 4:**
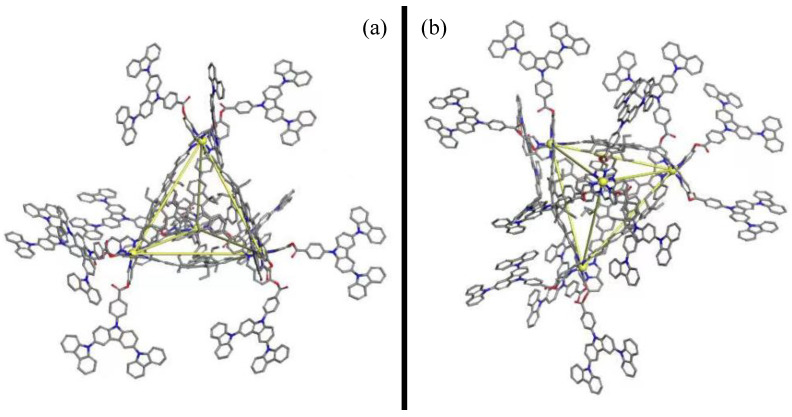
(**a**) Energy-minimized molecular structure of **Cage-1** (Zn, yellow; N, blue; O, red; C, gray). Hydrogens and counteranions are omitted for clarity ((**a**) side view and (**b**) top view of **Cage-1**).

Numerous efforts to obtain complete data for the single-crystal X-ray analysis of **Cage-1** were unsuccessful; therefore, a simulated molecular model of **Cage-1** was constructed (Figure 4a,b and Figure S18). The analysis of the simulated molecular model revealed that the structure of **Cage-1’s** supramolecular cage was exactly consistent with the expected tetrahedral architecture, in which the truxene units, dendritic carbazole arms, and Zn (II) centers constructed the faces, the extended ‘‘tails’’, and the vertices, respectively. Half of the ethyl chain of the benzene ring pointed into the cavity, while the rest pointed outward. The distance between any two adjacent zinc ions in the tetrahedron was 24.2 Å, as measured by Materials Studio software.

**Figure 5:**
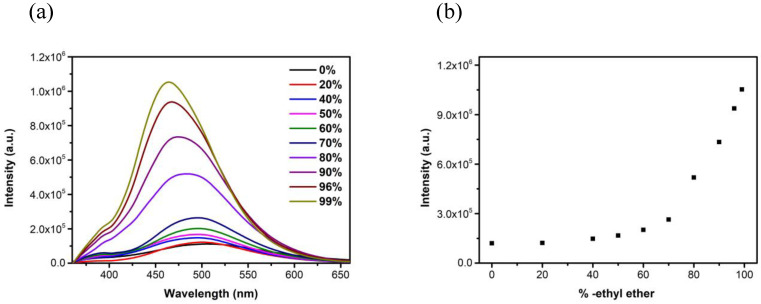
(**a**) Fluorescence emission spectra of ligand **LA** in varying proportions of chloroform–ethyl ether solvent mixture; (**b**) Fluorescence intensity of ligand **LA** at 340 nm in the varying proportions of chloroform–ethyl ether solvent mixture (c = 1.0 × 10^−6^ M, λ_ex_ = 340 nm, Slit = 3, 3).

Ligand **LB** has a solvent effect and aggregation-induced emission enhancement (AIEE) behavior due to its origin in the different solubility of the ligand in diverse solvents and different stacking modes in diverse solvents [50]. Carbazole derivatives are known to show a solvent influence on the difference in fluorescence color and good AIE behavior [38]. Thus, because of the twisted structure of ligand **LA,** which has a dendritic carbazole arm, it ought to hold a solvent-effect-induced fluorescence variation and AIE behavior as well. Consistent with expectations, similar phenomena were observed for ligand **LA**: the solvent effect caused spectral variations in its absorbance and fluorescence. Owing to electrons performing n-π* transitions in the large conjugated system, the UV–Vis absorption of ligand **LA** occurs at 280–350 nm (Figure S15a). The fluorescence emission spectra of ligand **LA** manifested diverse photoluminescence intensity and maximum emission wavelengths in different solvents, such as toluene, EA, chloroform, and 1,4-dioxane (Figure S15b). Additionally, the fluorescence spectra of ligand **LA** in different ratios of chloroform (desirable solvent) and ethyl ether (undesirable solvent) mixtures were examined. The photoluminescence intensity of ligand **LA** was gradually augmented, accompanied by an apparent hypochromatic shift of the maximum emission wavelength from 533 nm to 464 nm, along with an increasing fraction of ethyl ether in the chloroform—ethyl ether solvent mixture, showing good AIE behaviors (Figure 5a,b).

**Figure 6:**
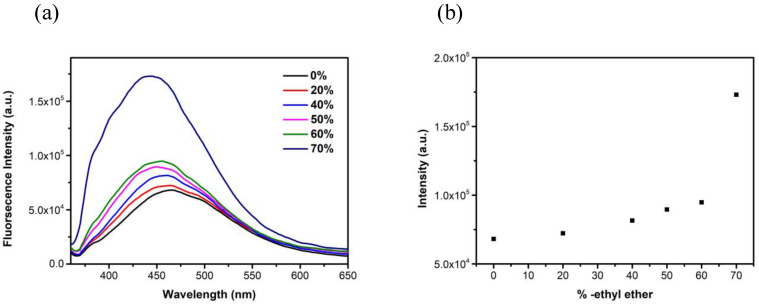
(**a**) Fluorescence emission spectra of **Cage-1** in the varying proportions of acetone–ethyl ether solvent mixture; (**b**) Fluorescence intensity of **Cage-1** at 340 nm in the varying proportions of acetone–ethyl ether solvent mixture (c = 1.0 × 10^−6^ M, λ_ex_ = 340 nm, Slit = 2, 2).

In consideration of the great solvent effect and AIE properties of ligands **LA** and **LB**, these two ligands were introduced to the supramolecular cage, and the fluorescence properties of **Cage-1** were subsequently investigated. Initially, the ultraviolet absorption spectrum of **Cage-1** was examined. The data obtained from the test showed that, compared with THF and acetone, the absorbance of methylene chloride and chloroform at 380–525 nm was significantly enhanced, indicating that nanometer aggregation occurred in these minor polar solvents. Then, **Cage-1** exhibited different photoluminescence effects in a limited number of benign solvents (THF, chloroform, DCM, and acetone), which also indicated that **Cage-1** had diverse fluorescence responses to diverse solvents (Figure S16b–d). **Cage-1** exhibited different fluorescence intensities in diverse fractions of an acetone (solubilizing solvent)–ethyl ether (insolubilizing solvent) mixture. As shown in Figure 6, when the percentage of ethyl ether was 0% to 60%, the fluorescence intensity of **Cage-1** showed a slow growth trend. Surprisingly, when the percentage of ethyl ether was increased to 70%, the fluorescence intensity of **Cage-1** suddenly increased by a factor of 2.5, which was believed to be caused by the restriction of intramolecular rotation (RIR). However, when the acetone/ethyl ether exceeded 70%, the cage began to precipitate gradually, such that the fluorescence data (the percentage of ethyl ether >70%) were inaccurate, which prevented us from exploring its unabridged AIE behavior. In brief, **Cage-1** showed a considerable solvent effect in a limited solvent and demonstrated the ability of aggregation-induced emission. Meanwhile, it also demonstrated great potential for tunable photoluminescent materials.

**Figure 7:**
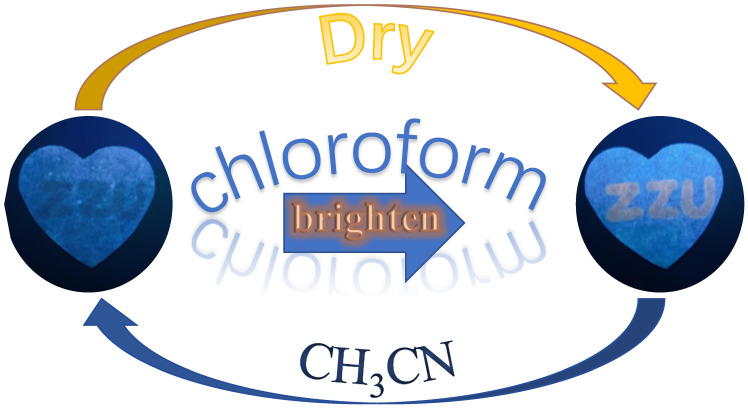
Vapor-responsive information recognition of **Cage-1**.

Considering that **Cage-1** had fluorescence-enhancement properties when the cage was aggregated, its application as a fluorescent ink was further studied. As illuminated in Figure 7, “ZZU” (Zhengzhou University) was written on the filter paper with a solution (chloroform: acetonitrile = 1:1) containing **Cage-1**. When the solvent had completely evaporated, “ZZU” was extremely hard to see on the filter paper, indicating that solid **Cage-1** did not exhibit fluorescence. Interestingly, after the filter paper was moistened with chloroform vapor, “ZZU” appeared as a bright, fluorescent orange color, and the orange fluorescence was still bright when the chloroform solvent had evaporated. Subsequently, when the filter paper was moistened again with acetonitrile steam, the fluorescence disappeared immediately. Importantly, “ZZU” reappeared when the wet filter paper became dry. The reversibility of the color could be achieved once more on this basis. Therefore, metal–organic supramolecular **Cage-1** may be used as an anticounterfeiting ink and applied in vapor-responsive information recognition.

## 3. Materials and Methods

### 3.1. Materials

All reagents were purchased from Sigma-Aldrich, Shanghai, China, Fisher, Shanghai, China, Across, Shanghai, China, and Alfa Aesar, Tianjin, China, and they were used without further purification. All solvents were dried according to standard procedures, and all of them were degassed under Ar for 30 min before use. All air-sensitive reactions were carried out under an inert Ar atmosphere.

### 3.2. Measurements

Column chromatography was conducted using SiO_2_ (VWR, 40–60 µm, 60 Å), and the separated products were visualized by UV light. NMR spectra data were recorded on a 600 MHz Bruker NMR spectrometer in CDCl_3_, DMSO, and CD_3_CN, with TMS as the reference. The UV–Vis spectra were recorded on a dual-beam UV–Vis spectrophotometer (TU-1901), PERSEE, Beijing, China. Emission spectra in the liquid state were recorded on a Horiba-FluoroMax-4 spectrofluorometer, HORIBA, Edison, NJ, USA; a 1 cm quartz cuvette was employed as the vessel for the recording of the fluorescence emission spectra. The crystal structure of ligand **LA** was recorded on a Rigaku XtaLAB Pro, Beijing, China. ESI-MS was recorded with a Waters Synapt G2-Si mass spectrometer, USA. High-resolution electrospray ionization mass spectrometry (HR-ESI-MS) experiments were performed with a Waters Q-Tof Micro MS/MS high-resolution mass, USA, spectrometer in ESI mode. Powder X-ray Diffraction (PXRD) was recorded on an X’Pert PRO, Nalytical,Almelo, Powder X-ray diffraction instrument. The Fourier Transform Infrared FT-IR spectra were recorded on a Spectrum TWO FT-IR spectrophotometer, PerkinElmer, Llantrisant, UK. The TGA was recorded on NETZSCH STA 2500, Germany.

### 3.3. Materials Synthesis

Compound **5** was synthesized according to the method in the literature [51].

#### 3.3.1. Preparation of Compound **5**

Compound **4** (497.19 mg 1.0 mmol), 1-iodo-4-methbenzoate (335.296 mg, 1.28 mmol), Cu_2_O (422.713 mg, 2.98 mmol), and DMAc (3 mL) were filled sequentially into a seal tube under a nitrogen atmosphere and heated to 160 °C in an oil bath for 24 h. Then, the mixture was cooled to room temperature and filtrated. The filtrate was poured into 60 mL H_2_O and stirred for 20 min. The crude products were collected by filtration and purified by chromatography (silica gel, petroleum ether/ethyl acetate, V:V = 10: 1) to give 322.2 mg (51%) of a white solid. m.p.: 253–254 °C. ^1^H NMR (600 MHz, CDCl_3_) δ 8.39 (d, J = 8.5 Hz, 2H), 8.28 (d, J = 1.8 Hz, 2H), 8.15 (d, J = 7.8 Hz, 4H), 7.84 (d, J = 8.5 Hz, 2H), 7.70 (d, J = 8.7 Hz, 2H), 7.63 (dd, J = 8.7, 2.0 Hz, 2H), 7.39 (dd, J = 3.6, 1.4 Hz, 8H), 7.27 (m, 4H), 4.01 (s, 3H). ^13^C NMR (151 MHz, CDCl_3_) δ 166.2, 141.7, 141.4, 140.1, 131.7, 131,0, 129.6, 126.6, 126.5, 125.9, 124.4, 123.2, 120.3, 119.8, 111.3, 109.6, 52.5. HRMS (ESI^+^, CHCl_3_) m/z: [M + H]^+^ calcd for C_44_H_29_N_3_O_2_: 632.2333; found: 632.2331.

#### 3.3.2. Preparation of Compound **6**

A mixture of Compound **5** (623.5 mg 0.99 mmol), 4.3 mL methanol, 8.6 mL THF, and 4.3 mL 0.8 M aqueous NaOH was stirred and refluxed for 8 h. After cooling to room temperature, the mixture was poured into 449 mL H_2_O and acidified by addition of 9.2 mL 35% HCl and stirred for 20 min. It was filtered to collect a white solid product: 348 mg (57%). m.p.: 287–289 °C. ^1^H NMR (600 MHz, DMSO) δ 13.23 (s, 1H), 8.72 (s, 2H), 8.29 (dd, J = 42.7, 6.7 Hz, 6H), 8.02 (d, J = 6.7 Hz, 2H), 7.76 (dd, J = 64.1, 7.5 Hz, 4H), 7.38 (dd, J = 48.1, 38.0 Hz, 12H). ^13^C NMR (151 MHz, DMSO) δ 167.2, 162.8, 141.5, 140.9, 140.0, 132.0, 131.0, 127.1, 126.7, 126.6, 124.6, 123,0, 121.0, 121.0, 120.2, 112.0, 110.2. HRMS (ESI^+^, CHCl_3_) m/z: [M + H]^+^ calcd for C_43_H_27_N_3_O_2_: 618.2179; found: 618.2176.

#### 3.3.3. Preparation of Ligand **LA**

Compound **6** (154.00 mg, 0.25 mmol), Compound **7** (44.8 mg, 0.36 mmol), dicyclohexylcarbodiimide (154.75 mg, 0.75 mmol), and 4-dimethylaminopyridine (9.16 mg, 0.075 mmol) were added to a Schlenk flask. After the removal of air and backfilling with argon, 5 mL dichloromethane was added. After being stirred at room temperature for 8 h, the solvent was removed under reduced pressure and purified by chromatography (silica gel, dichloromethane/petroleum ether, V: V = 10:1) to give 122.0 mg (68%) of a light-yellow solid. m.p.: 181–183 °C. ^1^H NMR (600 MHz, CDCl_3_) δ 10.13 (s, 1H), 8.82 (d, J = 2.3 Hz, 1H), 8.58 (d, J = 8.3 Hz, 2H), 8.31 (s, 2H), 8.15 (dd, J = 17.3, 8.1 Hz, 5H), 7.98 (d, J = 8.3 Hz, 2H), 7.90 (dd, J = 8.4, 2.3 Hz, 1H), 7.77 (d, J = 8.7 Hz, 2H), 7.67 (d, J = 8.6 Hz, 2H), 7.40 (d, J = 5.8 Hz, 7H), 7.31–7.27 (m, 4H).^13^C NMR (151 MHz, CDCl_3_) δ 192.0, 163.3, 150.6, 150.4, 144.0, 142.8, 141.7, 139.8, 132.6, 131.3, 130.1, 127.3, 126.9, 126.6, 126.0, 124.7, 123.3, 122.7, 120.4, 119.9, 111.3, 109.6.

#### 3.3.4. Preparation of **Cage-1**

Zn(NTf_2_)_2_ (7.5 mg, 12 μ mol, 1.2 equiv) was added to a solution of ligand **LB** (7.8 mg, 10 μ mol, 1.0 equiv) and ligand **LA** (22.4 mg, 31 μ mol, 3.1 equiv) in acetone (3.0 mL), and the whole reaction mixture was stirred at room temperature for 8 h and cooled at room temperature. The reaction mixture was poured into 4.5 mL ether to produce a precipitate. The precipitate was collected by centrifugation. The precipitate was washed twice with acetone: ether (V: V = 1: 2). Then, an orange solid cage, **Cage-1,** (29.4 mg, 21%) was obtained. ^1^H NMR (600 MHz, CD_3_CN) δ 9.23 (s, 1H), 8.64 (d, J = 8.1 Hz, 1H), 8.55 (d, J = 7.9 Hz, 1H), 8.46 (d, J = 7.9 Hz, 2H), 8.26 (s, 2H), 8.05 (t, J = 9.9 Hz, 6H), 7.84 (d, J = 21.3 Hz, 2H), 7.60–7.57 (m, 2H), 7.55 (s, 2H), 7.50 (d, J = 7.4 Hz, 2H), 7.42 (d, J = 8.3 Hz, 2H), 7.25−7.08 (m, 14H), 3.01 (s, 1H), 2.82 (s, 1H), 1.63 (s, 1H), 0.22 (s, 3H), −1.05 (s, 3H).

## 4. Conclusions

In summary, tetrahedral metal–organic supramolecular **Cage-1** with 12 dendritic carbazole arms was resoundingly constructed through self-assembly driven by coordination, followed by detailed ^1^H NMR and ESI-MS spectrometry characterization. Owing to the tunable fluorescence rendered by the dendritic carbazole arms and truxene-based amine, **Cage-1** was confirmed as exhibiting the potential for AIE behavior, accompanied by an interesting solvatochromic fluorescent behavior. Fundamentally, **Cage-1** could be used as anticounterfeiting, fluorescent ink for the hiding and identification of vapor-response information. This study enriches our insight into supramolecular chemistry involving AIE and the development of luminescent materials.

## Data Availability

Not applicable.

## Supplementary Materials

The supporting information can be downloaded at: https://www.mdpi.com/article/10.3390/ijms232415580/s1. References [52,53] are cited in Supplementary Materials.
