# Supplementary material for: Synthesis of a Tetrahedral Metal–Organic Supramolecular Cage with Dendritic Carbazole Arms
Source: Int J Mol Sci. 2022 Dec 8;23(24):15580. doi: 10.3390/ijms232415580 (PMC9779595; doi:10.3390/ijms232415580)
Supplement: Supplementary file 1 [file ijms-23-15580-s001.zip › ijms-2040761-supplementary.pdf]

Supporting Information

1 General Procedures

All reagents were purchased from Sigma-Aldrich, Fisher, Across, and Alfa Aesar, and were used without further purification. All solvents were dried according to standard procedures and all of them were degassed under Ar for 30 minutes before use. All air-sensitive reactions were carried out under an inert Ar atmosphere. Compounds **1**, **2**, **3**, **4**, and **5** were synthesized according to the literature method[1,2]. Compound **9** was synthesized according to a literature method[3]. Column chromatography was conducted using SiO_2 (VWR, 40-60 µm, 60 Å) and the separated products were visualized by UV light. NMR spectra data were recorded on a 600 MHz Bruker NMR spectrometer in $CDCl_3$, DMSO, and CD_3CN with TMS as the reference. The UV-vis spectra were recorded on a dual-beam UV-Vis spectrophotometer (TU-1901). Emission spectra in the liquid state were recorded on a Horiba-FluoroMax-4 spectrofluorometer, a 1 cm quartz cuvette was employed as the vessel for the recording of the fluorescence emission spectrum. The crystal structure of ligand **L-A** was recorded on a Rigaku XtaLAB Pro. Powder X-Ray Diffraction (PXRD) was recorded on an X'Pert PRO Powder X-ray diffraction instrument. The Fourier Transform Infrared FT-IR spectra were recorded on a Spectrum TWO FT-IR spectrophotometer.

ESI-MS and HR-ESI MS. ESI-MS was recorded with a Waters Synapt G2 -Si mass spectrometer. High-resolution electrospray ionization mass spectrometry (HR-ESI MS) experiments were performed with a Water Q-Tof Micro MS/MS high resolution mass spectrometer in ESI mode.

Thermogravimetric analysis (TGA). The TGA was recorded on NETZSCH STA 2500. The samples were heated at the rate of 10 °C/min to 900 °C under a nitrogen flow. All solid materials were desolvatedin a vacuum 2 hours prior to TGA analysis.

2 Synthetic experimental details and characterizations of Ligand L-A and Ligand L-B

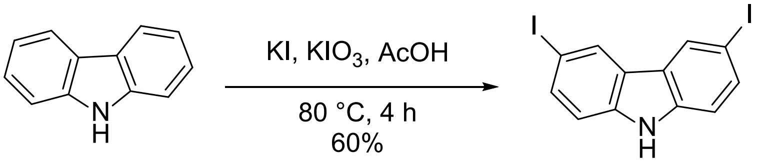

1

Synthesis of compound **1**.

Carbazole (3.76 g, 22.52 mmol) was dissolved in AcOH (152 mL) and then heated to 80 ℃, and reflux for 30 minutes, then KIO_3 (3.64 g, 17.04 mmol), KI (5.01 g, 30.2 mmol) were added to react for 4 hours. The mixture was cooled to room temperature and filtrated. The filtrate was poured into a 5% $Na_2S_2O_3$ solution. The crude products were collected by filtration and recrystallized from dichloromethane to give 5.66 g (60%) of a light brown solid. m.p.: 211-212 ℃.^{1}H NMR (600 MHz, DMSO) δ 11.55 (s, 1H), 8.57 (s, 2H), 7.65 (dd, J = 21.5, 8.1 Hz, 2H), 7.35 (d, J = 8.5 Hz, 2H).

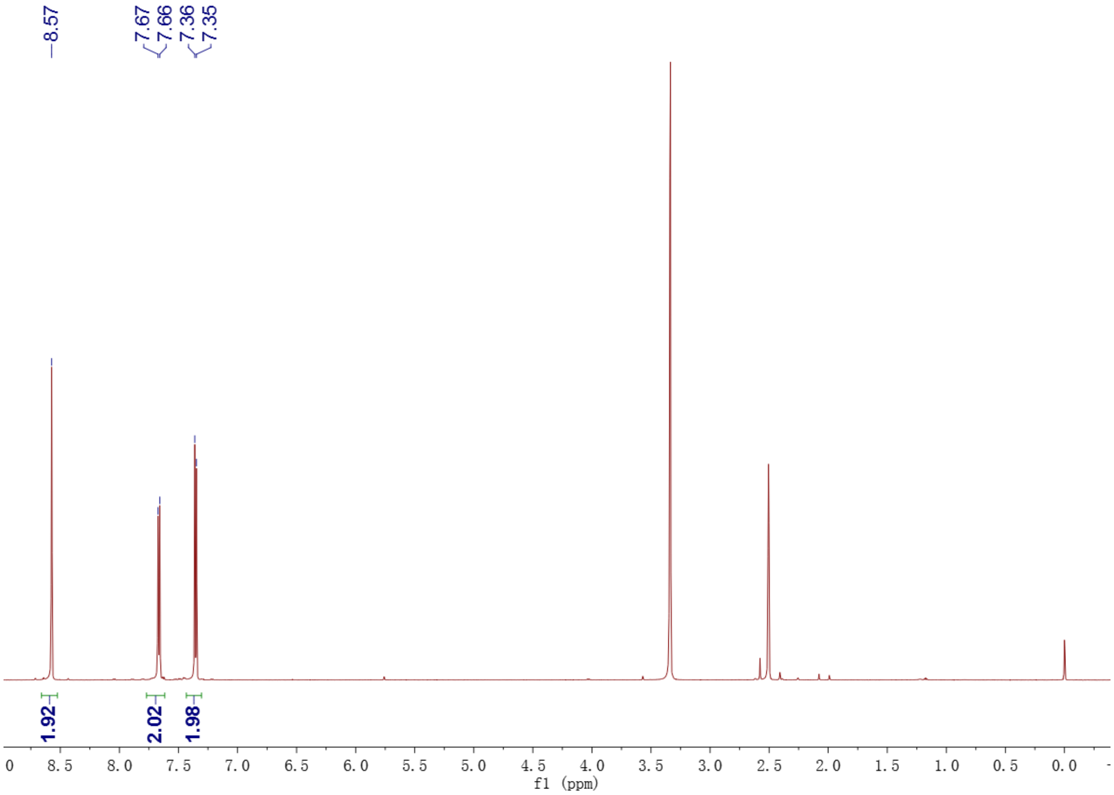

Figure S1. ^{1}H NMR (600 MHz, 298K, DMSO) spectrum of **1**.

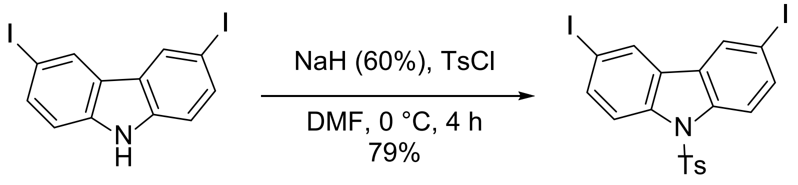

Synthesis of compound **2**.

Prepare a solution of 3,6-diiodo-9H-carbazole (2.5132 g, 6 mmol) in *N,N*-dimethylformamide (10 mL). Add NaH (575.52 mg, 24 mmol) to the above solution at 0 °C. Stir the above mixture for 10 minutes. Subsequently add TsCl (3.99 g, 21 mmol) to the mixture and stir at 0 °C for 4 hours. Dilute the mixture with water and filter the precipitate. Recrystallize the crude product from dichloro-methane to obtain 2.74 g (79%) compound **2**. m.p.: >250 °C. ^{1}H NMR (600 MHz, DMSO) δ 8.64 (d, J = 1.4 Hz, 2H), 8.05 (d, J = 8.8 Hz, 2H), 7.90 (d, J = 1.6 Hz, 2H), 7.73 (d, J = 8.4 Hz, 2H), 7.30 (d, J = 8.2 Hz, 2H), 2.51 (s, 3H).

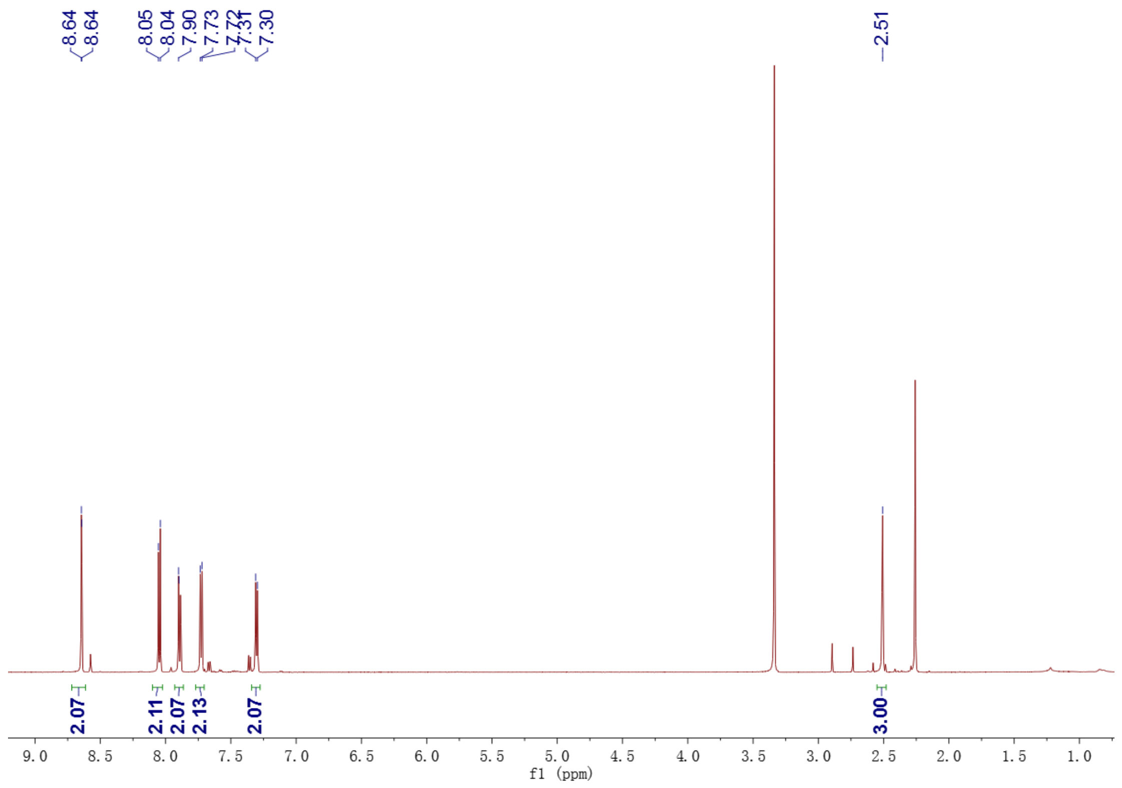

Figure S2. ^{1}H NMR (600 MHz, 298K, DMSO) spectrum of **2**.

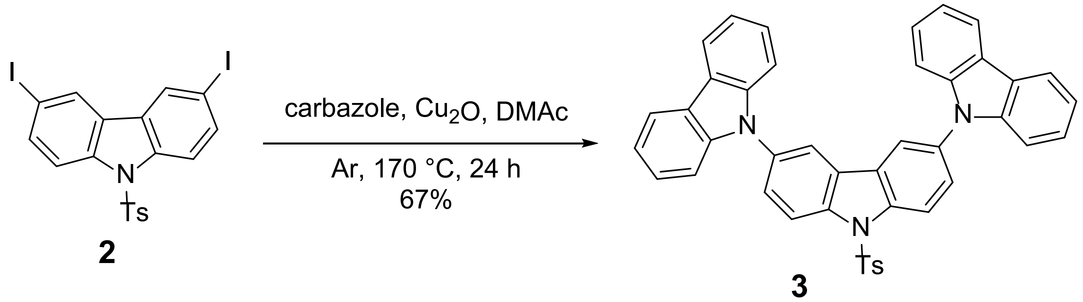

Synthesis of compound **3** (Ullmann reaction).

Carbazole (0.95 g, 5.68 mmol), compound **2** (1.3 g, 2.27 mmol), Cu_2O (1.13 g, 7.96 mmol), and DMAc (5 mL) were filled sequentially into a seal-tube under argon atmosphere and heated to 180 °C in oil bath for 24 h. Then, the mixture was cooled to room temperature and filtrated. The filtrate was poured into 100 mL H_2O and stirred for 20 min. The crude products were collected by filtration, and recrystallized from EtOH: THF (V: V = 1: 1,) to give 1.37 g (69 %) of a white solid. ^{1}H NMR (600 MHz, DMSO) δ 8.67 (s, 2H), 8.59 (d, J = 8.8 Hz, 2H), 8.25 (d, J = 7.7 Hz, 4H), 8.03 (d, J = 8.0 Hz, 2H), 7.87 (d, J = 8.9 Hz, 2H), 7.46 (d, J = 8.0 Hz, 2H), 7.41 (d, J = 3.4 Hz, 8H), 7.29 (dd, J = 7.4, 3.7 Hz, 4H), 2.35 (s, 3H).

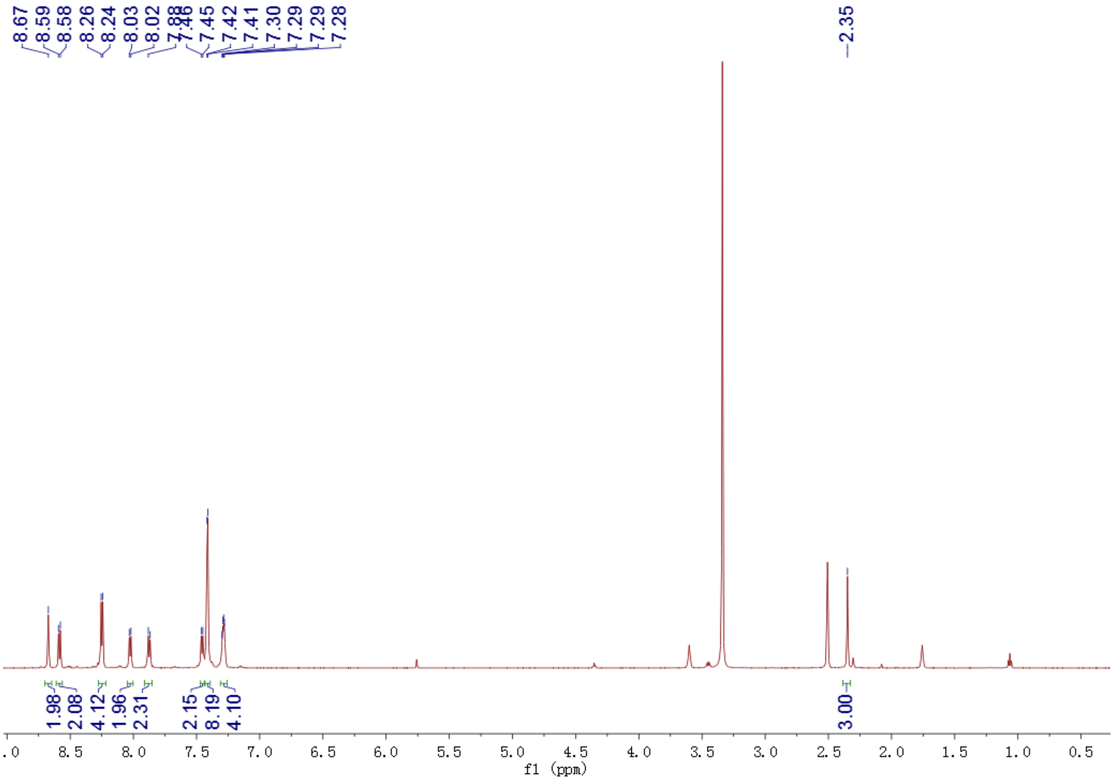

Figure S3. ^{1}H NMR (600 MHz, 298K, DMSO) spectrum of **3**.

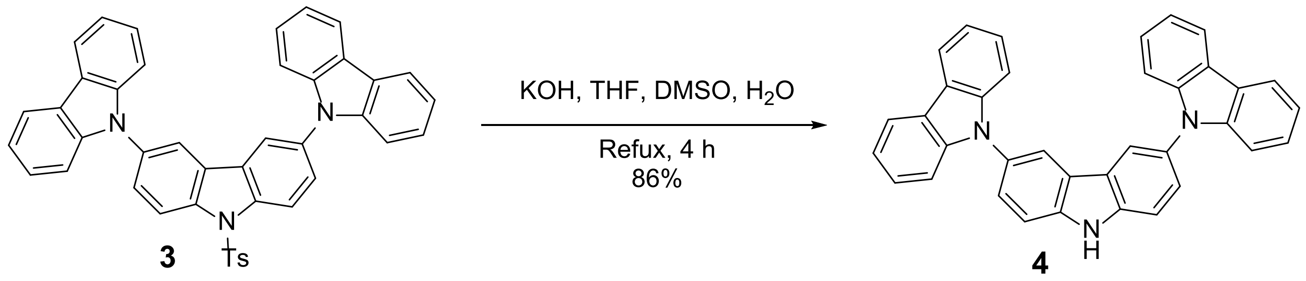

Synthesis of compound **4**.

Compound **3** (2.735 g, 3.12 mmol) was dissolved in THF (7.5 mL), DMSO (3.8 mL), and H_2O (1.5 mL), then KOH (3.492 g, 62.4 mmol) was added. The mixture was refluxed for 4 h (monitored by TLC). Then, cooled to room temperature, neutralized by HCl, and then poured into water to give the brown solid, which was recrystallized from EtOH: THF (V: V = 4: 1,) to give 1.54 g (86%) of a white solid. m.p.: ＞300 °C. ^{1}H NMR (600 MHz, DMSO) δ 11.90 (s, 1H), 8.51 (s, 2H), 8.25 (s, 4H), 7.85 (s, 2H), 7.62 (s, 2H), 7.44 – 7.24 (m, 12H).

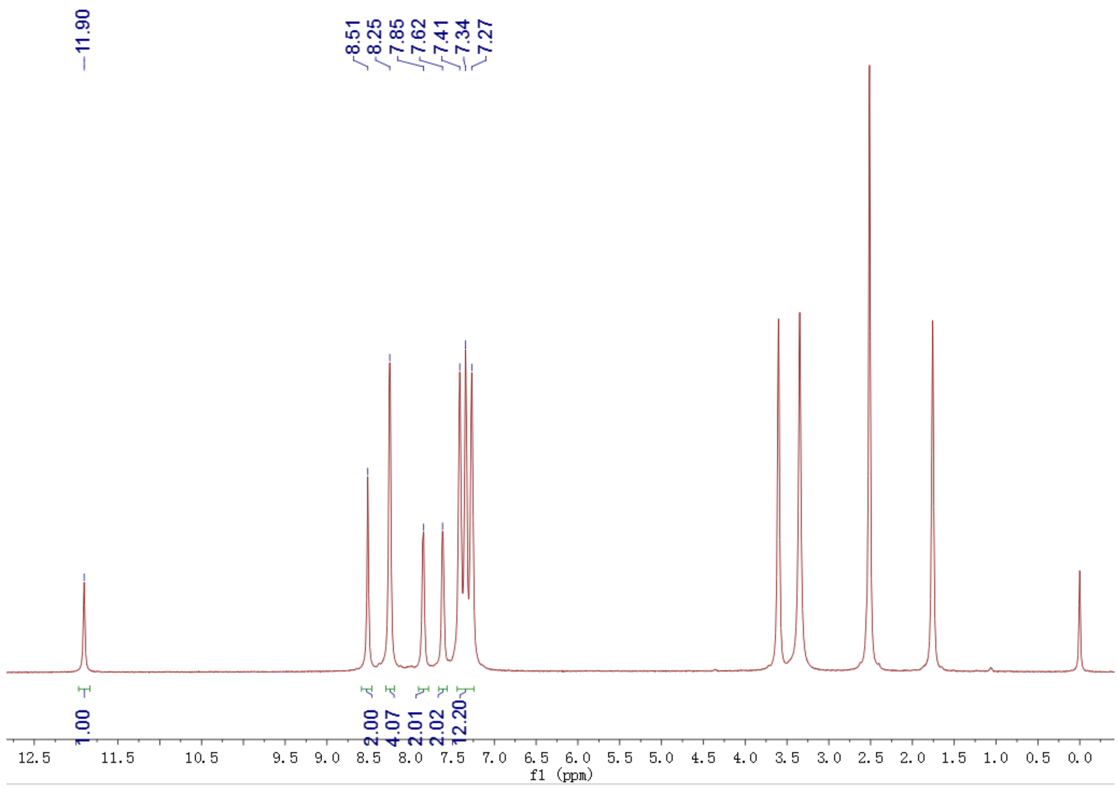

Figure S4. ^{1}H NMR (600 MHz, 298K, DMSO) spectrum of **4**.

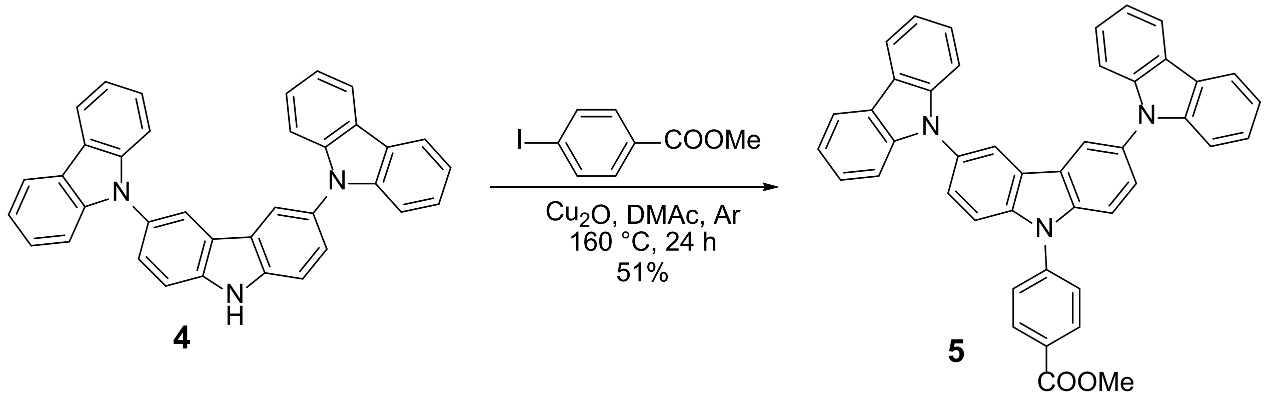

Synthesis of compound **5** (Ullmann reaction).

Compound **4** (497.19 mg 1.0 mmol), 1-iodo-4-methbenzoate (335.296 mg, 1.28 mmol), Cu_2O (422.713 mg, 2.98 mmol), and DMAc (3 mL) were filled sequentially into a seal-tube under nitrogen atmosphere and heated to 160 °C in oil bath for 24 h. Then, the mixture was cooled to room temperature and filtrated. The filtrate was poured into 60 mL H_2O and stirred for 20 min. The crude products were collected by filtration and purified by chromatography (silica gel, petroleum ether/ethyl acetate, V: V = 10: 1) to give 322.2 mg (51%) of a white solid. m.p.: 253-254 °C. ^{1}H NMR (600 MHz, $CDCl_3$) δ 8.39 (d, J = 8.5 Hz, 2H), 8.28 (d, J = 1.8 Hz, 2H), 8.15 (d, J = 7.8 Hz, 4H), 7.84 (d, J = 8.5 Hz, 2H), 7.70 (d, J = 8.7 Hz, 2H), 7.63 (dd, J = 8.7, 2.0 Hz, 2H), 7.39 (dd, J = 3.6, 1.4 Hz, 8H), 7.27 (m, 4H), 4.01 (s, 3H).^{13}C NMR (151 MHz, $CDCl_3$) δ 166.2, 141.7, 141.4, 140.1, 131.7, 131,0, 129.6, 126.6,

126.5, 125.9, 124.4, 123.2, 120.3, 119.8, 111.3, 109.6, 52.5. HRMS (ESI$^+$, $CHCl_3$) *m/z*: $[M+H]^+$ calcd for $C_{44}H_{29}N_3O_2$: 632.2333; found: 632.2331.

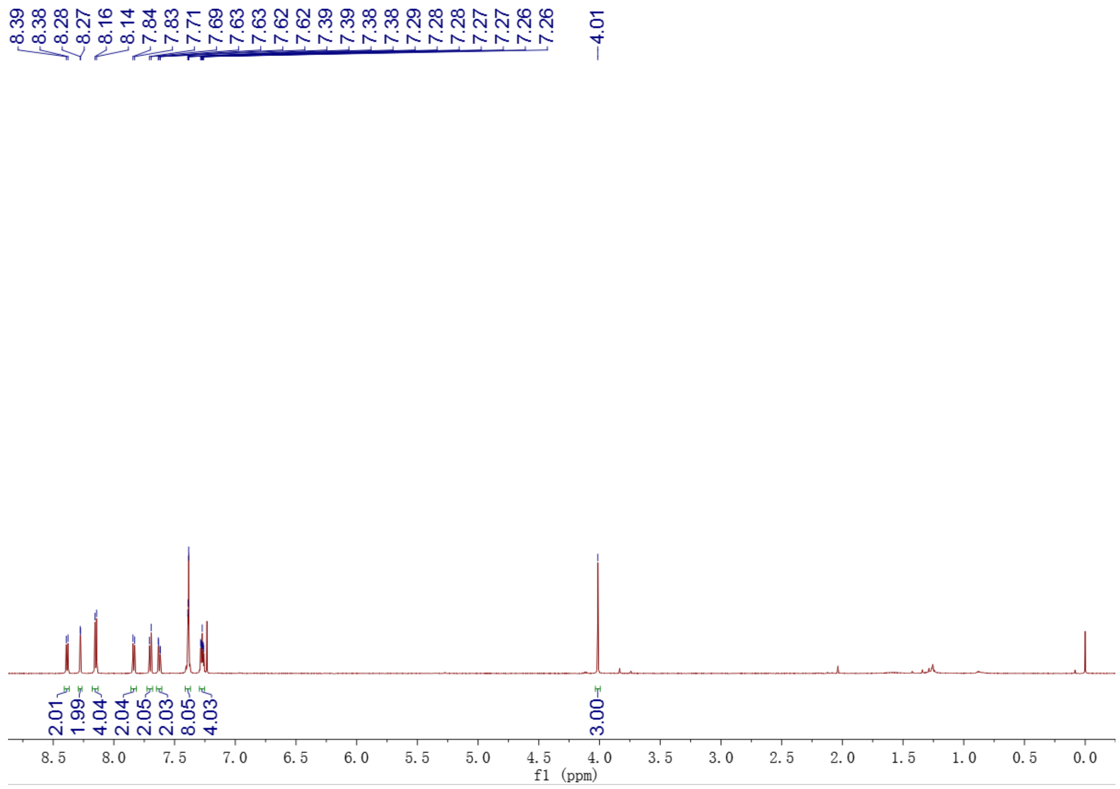

Figure S5. ^1H NMR (600 MHz, 298 K, $CDCl_3$) spectrum of **5**.

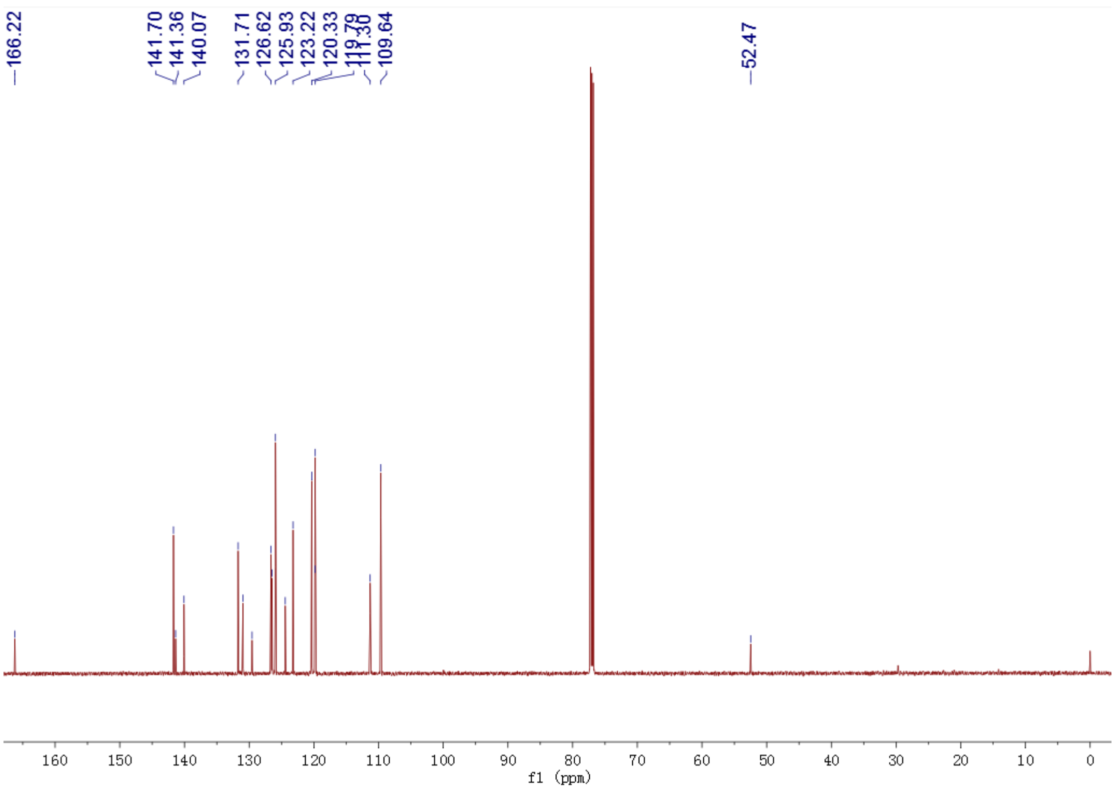

Figure S6. ^{13}C NMR (151 MHz, 298 K, $CDCl_3$) spectrum of **5**.

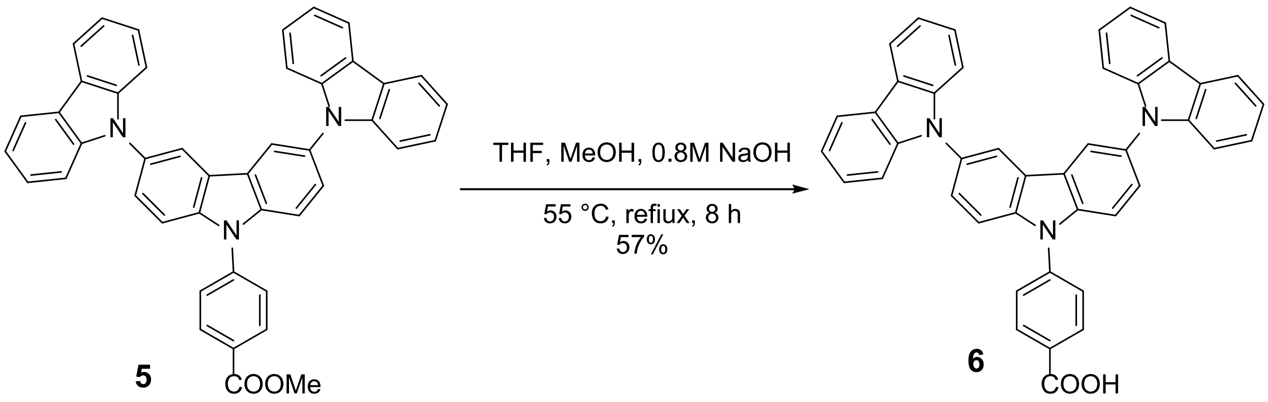

Synthesis of compound **6**.

A mixture of compound **5** (623.5 mg 0.99 mmol), 4.3 mL of methanol, 8.6 mL THF, and 4.3 mL of 0.8 M aqueous NaOH was stirred and refluxed for 8 h. After cooling to room temperature, the mixture was poured into 449 mL H_2O and acidified by addition of 9.2 mL of 35% HCl and stirred for 20 min. Filter to collect white solid product 348 mg (57%). m.p.: 287-289 °C. ^{1}H NMR (600 MHz, DMSO) δ 13.23 (s, 1H), 8.72 (s, 2H), 8.29 (dd, J = 42.7, 6.7 Hz, 6H), 8.02 (d, J = 6.7 Hz, 2H), 7.76 (dd, J = 64.1, 7.5 Hz, 4H), 7.38 (dd, J = 48.1, 38.0 Hz, 12H). ^{13}C NMR (151 MHz, DMSO) δ 167.2, 162.8, 141.5, 140.9, 140.0, 132.0, 131.0, 127.1, 126.7, 126.6, 124.6, 123,0, 121.0, 121.0, 120.2, 112.0, 110.2. HRMS (ESI$^+$, $CHCl_3$) m/z: $[M+H]^+$ calcd for $C_{43}H_{27}N_3O_2$: 618.2179; found: 618.2176.

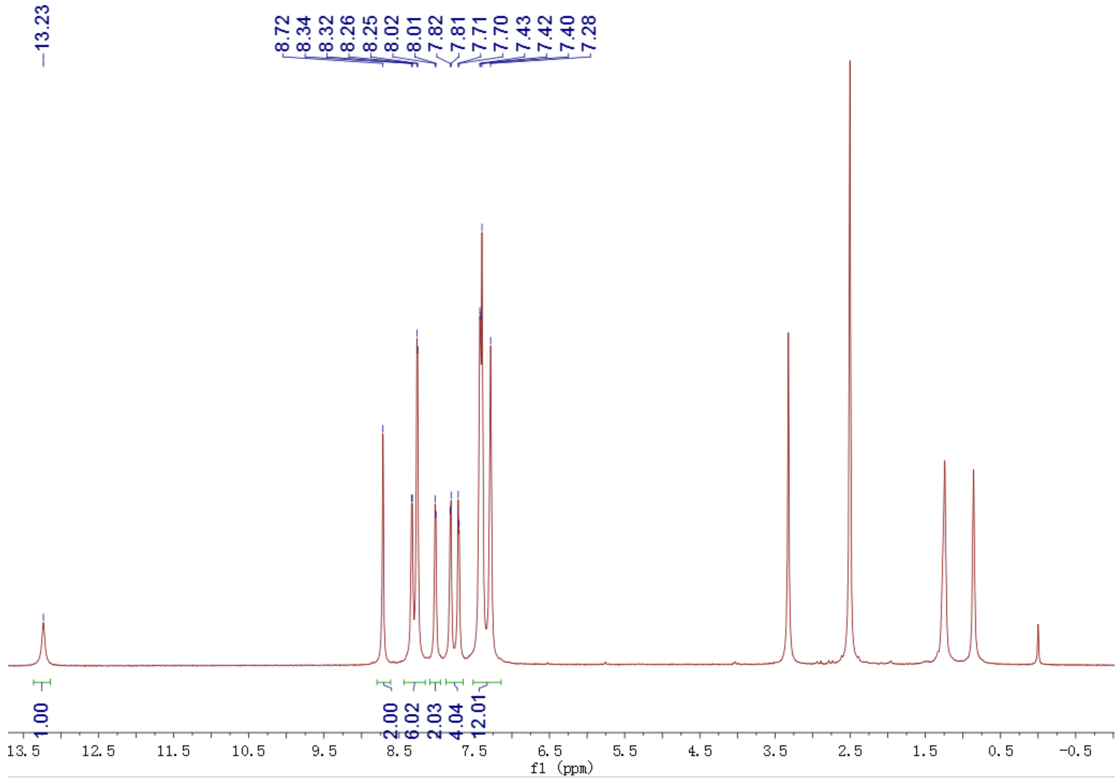

Figure S7. ^{1}H NMR (600 MHz, 298 K, DMSO) spectrum of **6**.

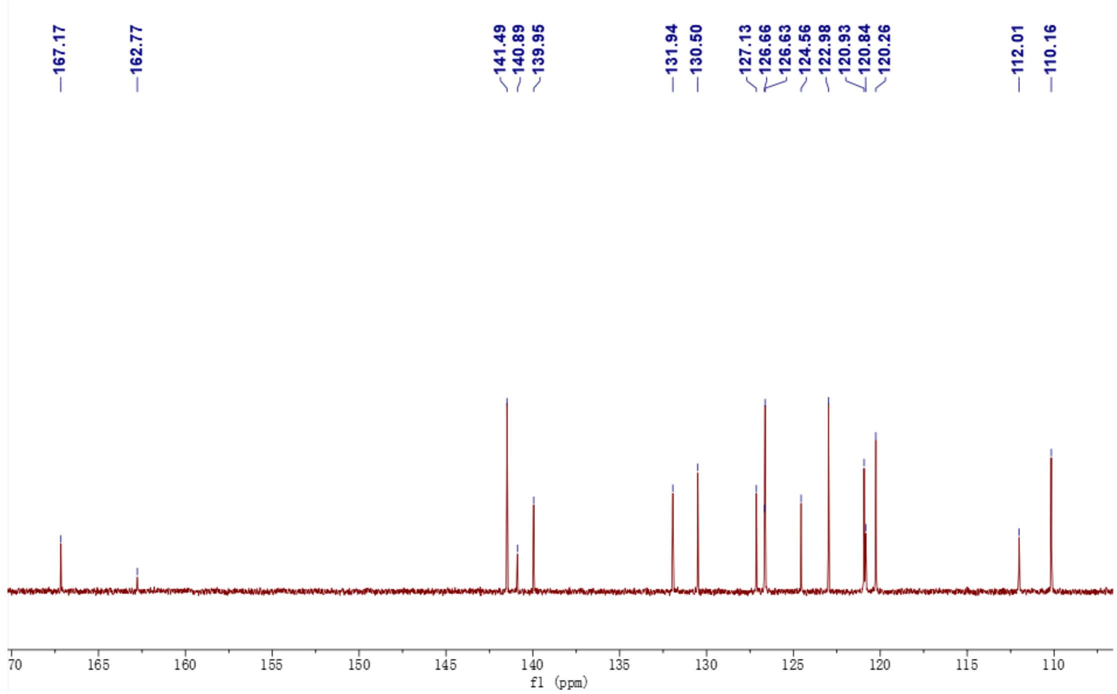

Figure S8. ^{13}C NMR (151 MHz, 298 K, DMSO) spectrum of **6**.

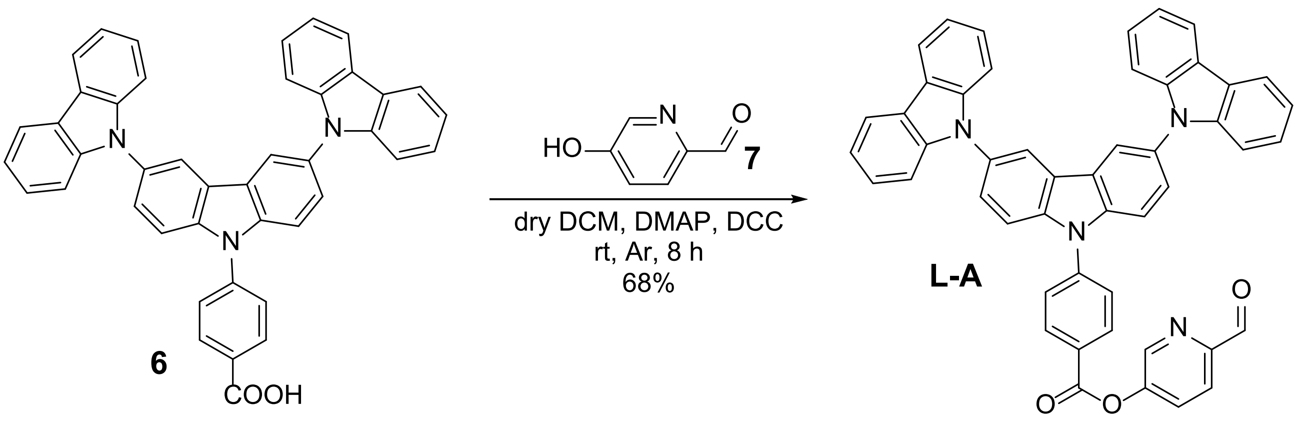

Synthesis of ligand **L-A**.

Compound **6** (154.00 mg, 0.25 mmol), Compound **7** (44.8 mg, 0.36 mmol), dicyclohexylcarbodiimide (154.75 mg, 0.75 mmol), and 4-dimethylaminopyridine (9.16 mg, 0.075 mmol) were added to a Schlenk flask. After the removal of air and back-filled with argon, 5 mL of dichloromethane was added. After stirred at room temperature for 8 h, the solvent was removed under reduced pressure and purified by chromatography (silica gel, dichloromethane/petroleum ether, V: V = 10: 1) to give 122.0 mg (68%) of a light yellow solid. m.p.: 181-183 °C. ^{1}H NMR (600 MHz, $CDCl_3$) δ 10.13 (s, 1H), 8.82 (d, J = 2.3 Hz, 1H), 8.58 (d, J = 8.3 Hz, 2H), 8.31 (s, 2H), 8.15 (dd, J = 17.3, 8.1 Hz, 5H), 7.98 (d, J = 8.3 Hz, 2H), 7.90 (dd, J = 8.4, 2.3 Hz, 1H), 7.77 (d, J = 8.7 Hz, 2H), 7.67 (d, J = 8.6 Hz, 2H), 7.40 (d, J = 5.8 Hz, 7H), 7.31 – 7.27 (m, 4H). ^{13}C NMR (151 MHz, $CDCl_3$) δ 192.0, 163.3, 150.6, 150.4, 144.0, 142.8, 141.7, 139.8, 132.6, 131.3, 130.1, 127.3, 126.9, 126.6, 126.0, 124.7, 123.3, 122.7, 120.4, 119.9, 111.3, 109.6.

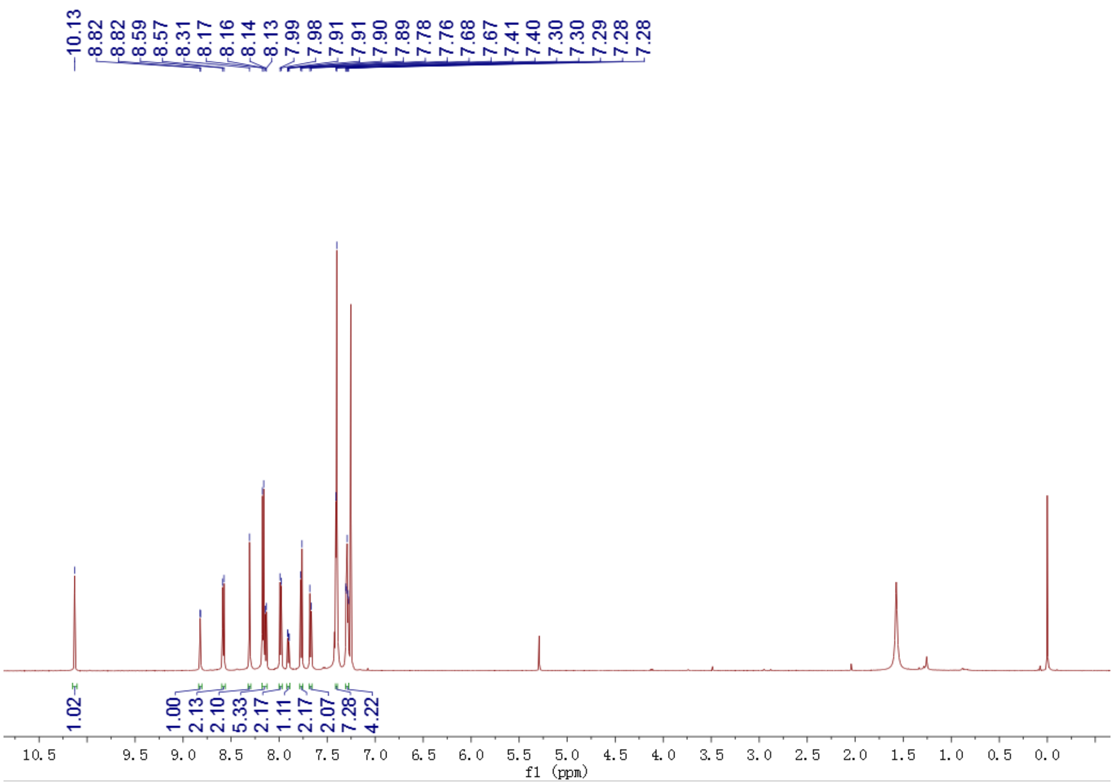

Figure S9. ^{1}H NMR (600 MHz, 298 K, $CDCl_3$) spectrum of **L-A**.

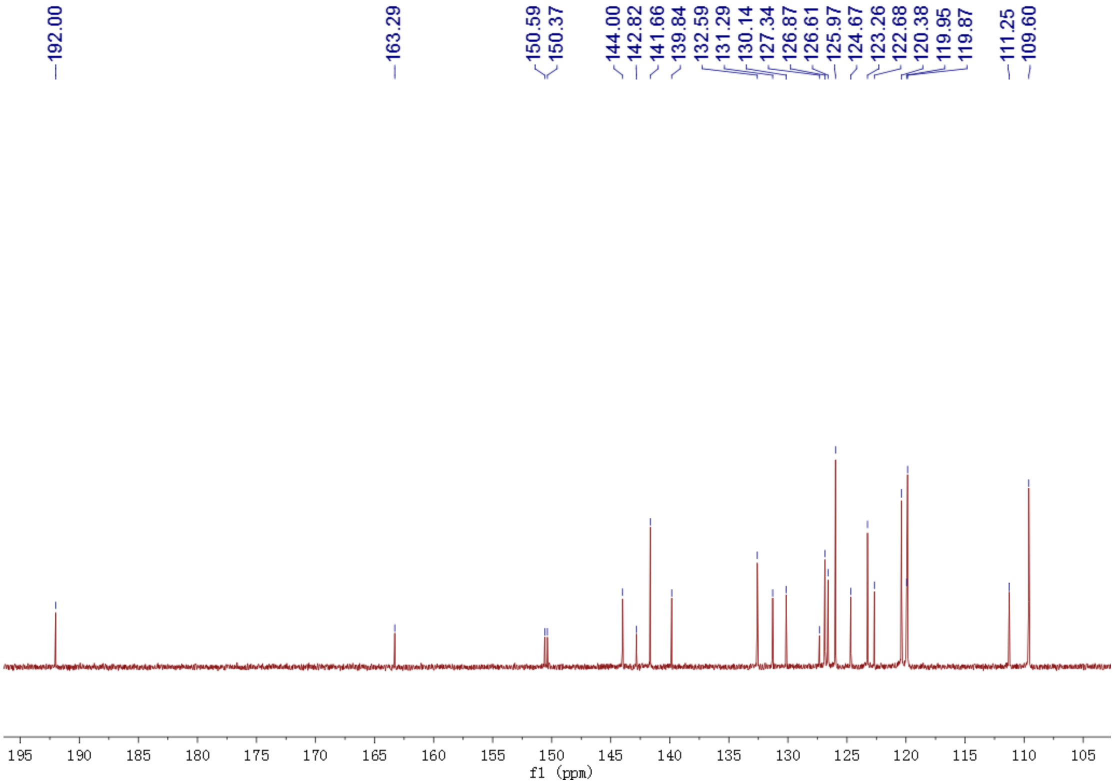

Figure S10. ^{13}C NMR (151 MHz, 298 K, $CDCl_3$) spectrum of **L-A**.

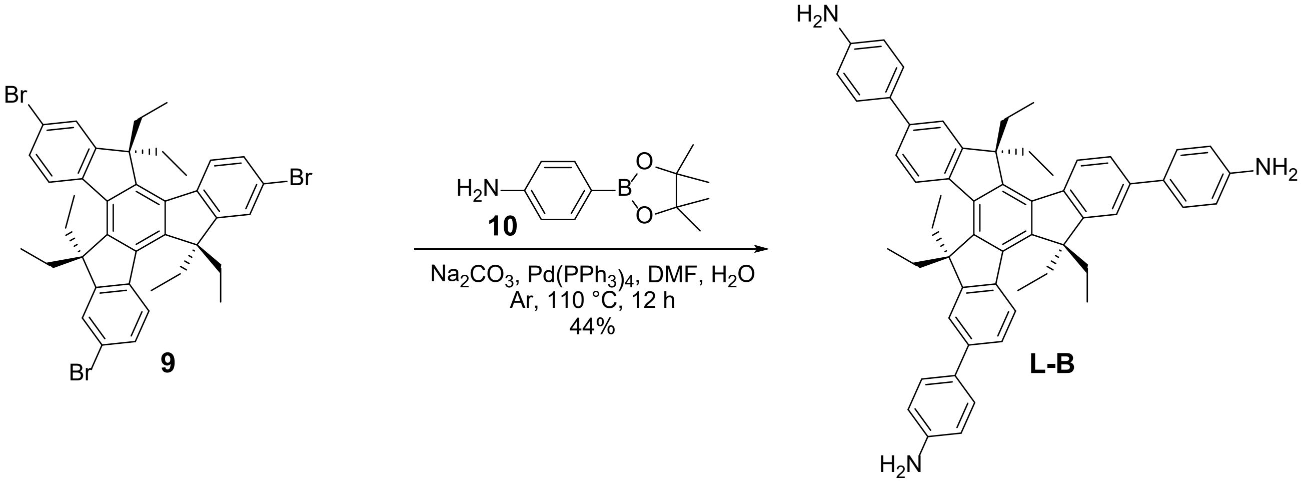

Synthesis of Ligand **L-B**.

To a solution of compound **10** (1.12 g, 1.5 mmol) in DMF: H_2O (40 ml, v: v=3: 1) was added 4-aminophenylboronic acid pinacol ester (1.08 g, 4.95 mmol), $Pd(PPh_3)_4$ (259.99 mg, 0.225 mmol), Na_2CO_3 (476.96 mg, 4.5 mmol) under an argon atmosphere. After stirring at 110 °C for 12 h, the reaction was cooled to room temperature. The mixture was extracted with ethyl acetate (3 × 30 mL), dried over anhydrous Na_2SO_4, filtered, and concentrated under reduced pressure. The residue was

purified by flash column chromatography (chloroform: methanol =100: 1) to give Ligand **L-B** (517.08 mg, 0.66 mmol) in 44% yield as a white solid. m.p.: 207-210 °C. ^{1}H NMR (600 MHz, $CDCl_3$) δ 8.37 (d, J = 8.2 Hz, 1H), 7.62 (d, J = 1.4 Hz, 1H), 7.60 (dd, J = 9.8, 1.5 Hz, 1H), 7.57 (d, J = 8.4 Hz, 2H), 6.82 (d, J = 8.3 Hz, 2H), 3.76 (s, 2H), 3.06 (dd, J = 13.8, 7.2 Hz, 2H), 2.21 (dd, J = 13.8, 7.3 Hz, 2H), 0.28 (t, J = 7.2 Hz, 6H). ^{13}C NMR (151 MHz, $CDCl_3$) δ 191.3, 140.3, 139.4, 139.1, 136.9, 131.0, 130.1, 129.9, 129.6, 126.1, 117.5, 114.8, 21.3. HR-ESI MS (m/z) calcd for $C_{57}H_{58}N_3^+$ $[M+H]^+$: 784.4625; found: 784.4621.

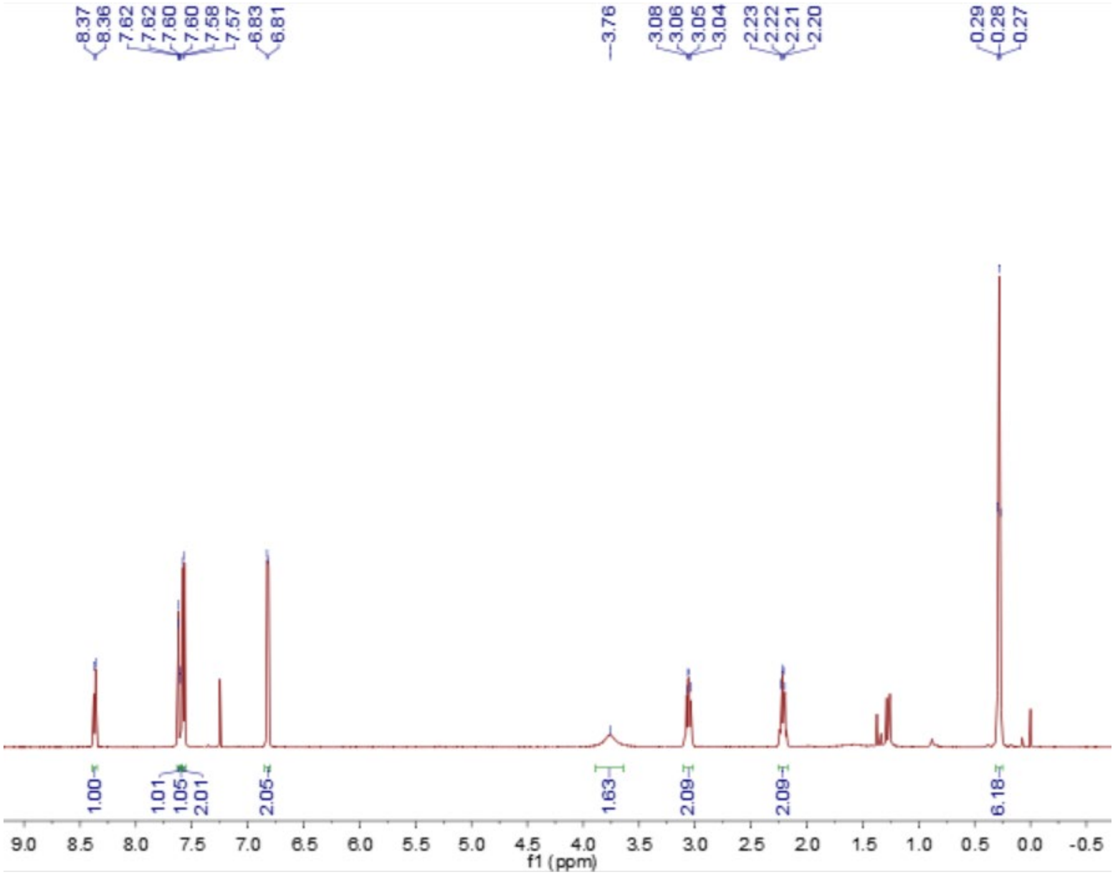

Figure S11. ^{1}H NMR (600 MHz, 298 K, $CDCl_3$) spectrum of **L-B**.

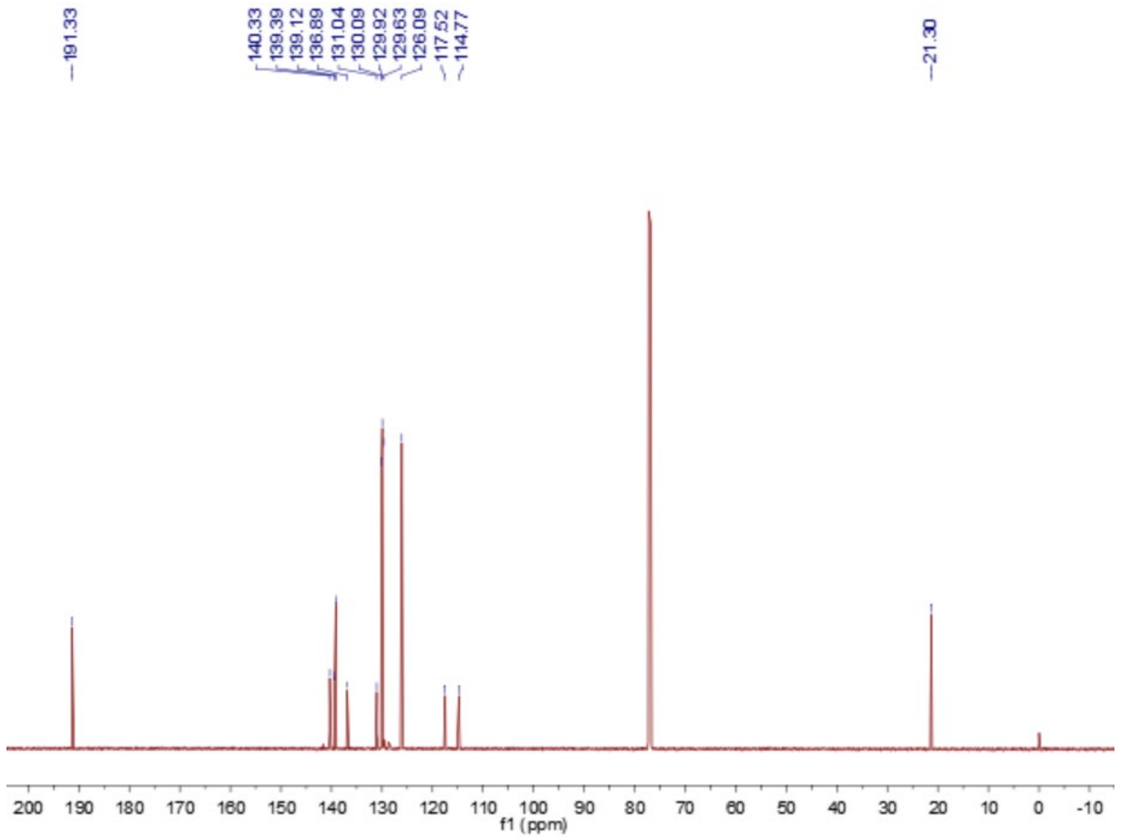

Figure S12. ^{13}C NMR (151 MHz, 298 K, $CDCl_3$) spectrum of **L-B**.

3 Synthetic experimental details and characterizations of Cage-1

Synthesis of **Cage-1**.

$Zn(NTf_2)_2$ (7.5 mg, 12 μ mol, 1.2 equiv) was added to a solution of Ligand **L-B** (7.8 mg, 10 μ mol, 1.0 equiv) and Ligand **L-A** (21.7 mg, 31 μ mol, 3.0 equiv) in acetone (3.0 mL), and the whole reaction mixture was stirred at room temperature for 8 h and cooled at room temperature. The reaction mixture was poured into 4.5 ml of ether to produce precipitate. The precipitate was collected by centrifugation. The precipitate was washed twice with Acetone: Ether (V: V = 1: 2). Then the orange solid cage **Cage-1**(29.4 mg, 21%) was obtained.^1H NMR (600 MHz, CD_3CN) δ 9.23 (s, 1H), 8.64 (d, J = 8.1 Hz, 1H), 8.55 (d, J = 7.9 Hz, 1H), 8.46 (d, J = 7.9 Hz, 2H), 8.26 (s, 2H), 8.05 (t, J = 9.9 Hz, 6H), 7.84 (d, J = 21.3 Hz, 2H), 7.60 – 7.57 (m, 2H), 7.55 (s, 2H), 7.50 (d, J = 7.4 Hz, 2H), 7.42 (d, J = 8.3 Hz, 2H), 7.25 – 7.08 (m, 14H), 3.01 (s, 1H), 2.82 (s, 1H), 1.63 (s, 1H), 0.22 (s, 3H), -1.05 (s, 3H).

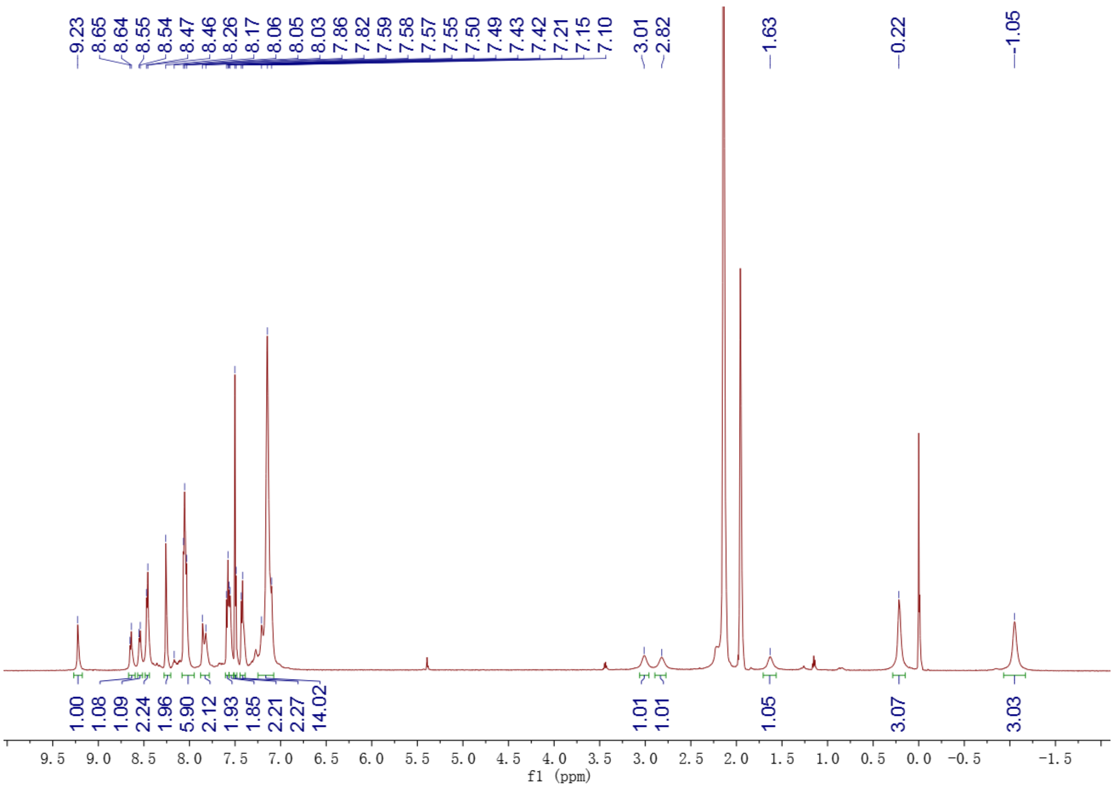

Figure S13. ^{1}H NMR (600 MHz, 298 K, $CDCl_3$: CD_3CN = 1: 1) spectrum of **Cage-1**.

4 ESI-MS data of Cage-1

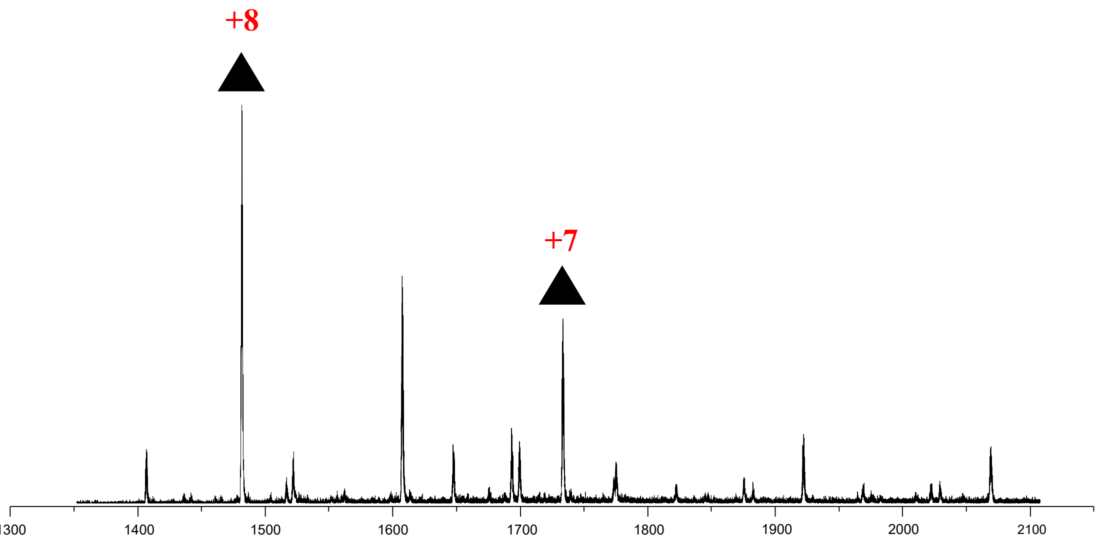

Figure S14. ESI-MS ($CDCl_3$: CD_3CN = 1: 1) of **Cage-1**.

5 Optical property data of Ligand L-A and Cage-1

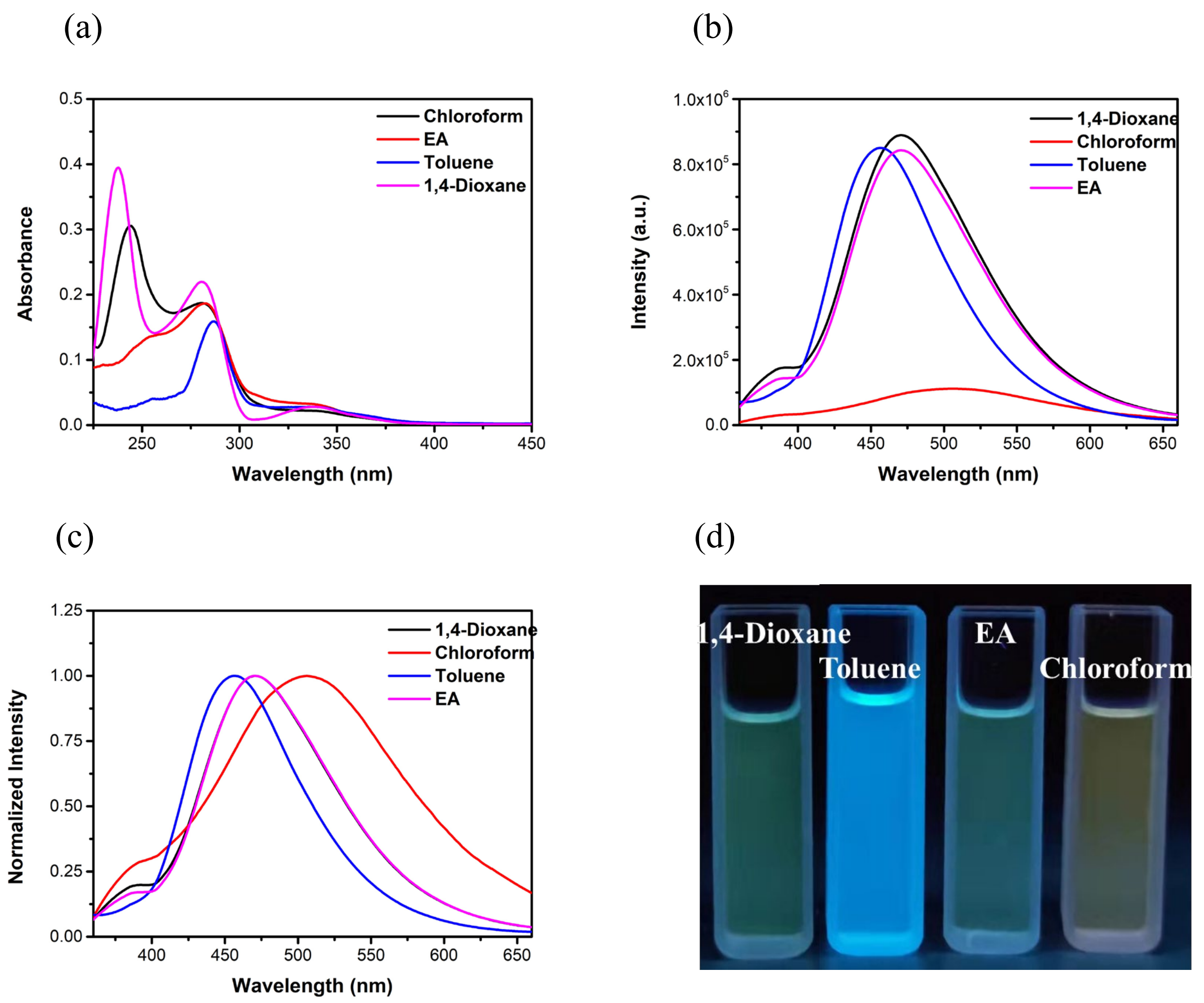

Figure S15. (a) UV-vis absorption spectra of ligand **L-A**, (b) Fluorescence spectra of ligand **L-A** (λ_{ex} = 340 nm, c = 1.0 μM, Slit = 3, 3.), (c) Normalized fluorescence emission spectra of ligand **L-A** and (d) photographs (c = 1 μM) under the 365 nm UV light of Ligand **L-A** in different solvents.

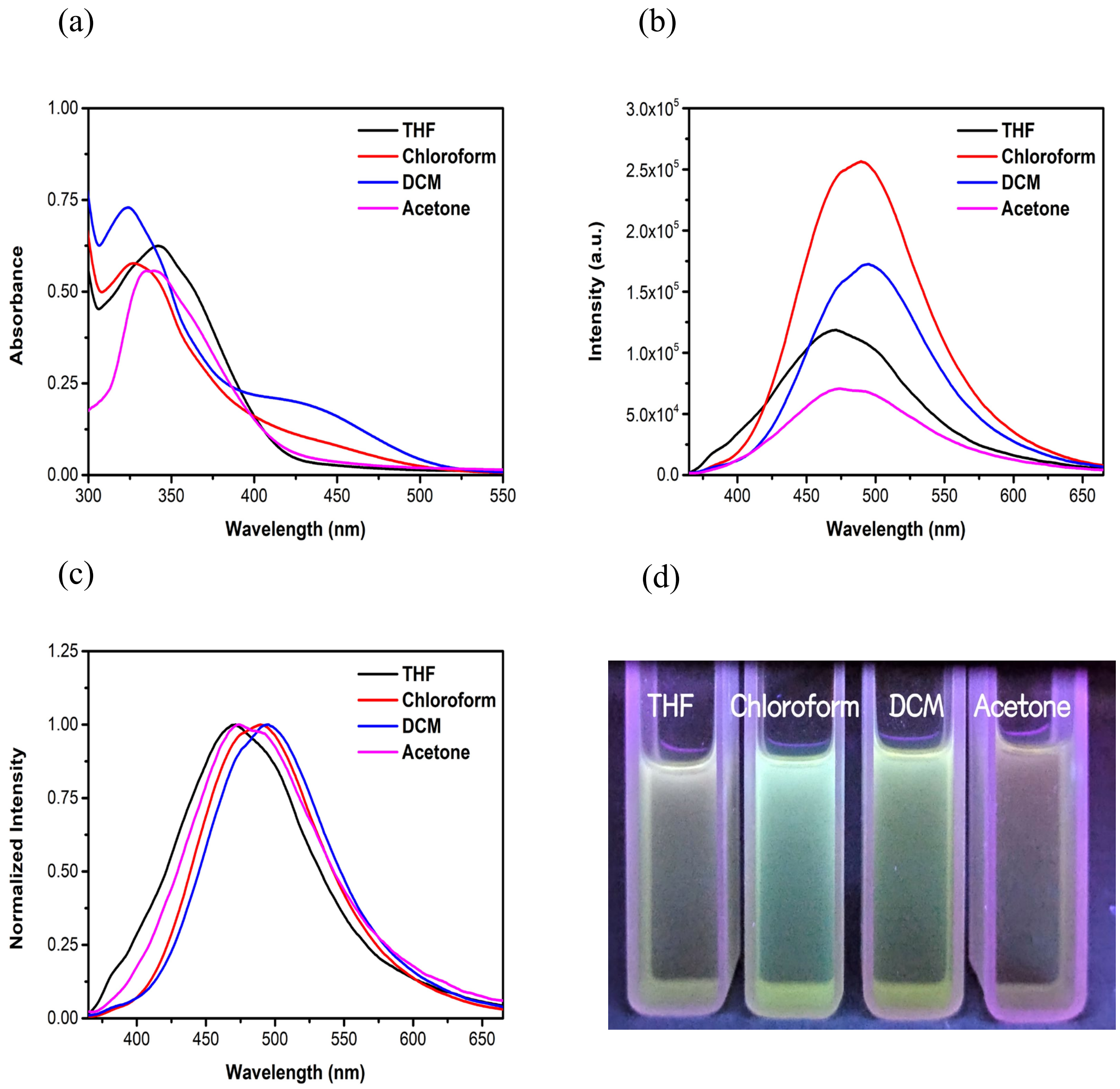

Figure S16. (a) UV-vis absorption spectra of **Cage-1**, (b) Fluorescence spectra of **Cage-1** (λ_{ex} = 342.5 nm, c = 1.0 × 10^{-6} M, Slit = 3, 3.), (c) Normalized fluorescence emission spectra of **Cage-1** and (d) photographs (c = 1 μM) under the 365 nm UV light of **Cage-1** in different solvents.

6 Crystal structure of ligand L-A

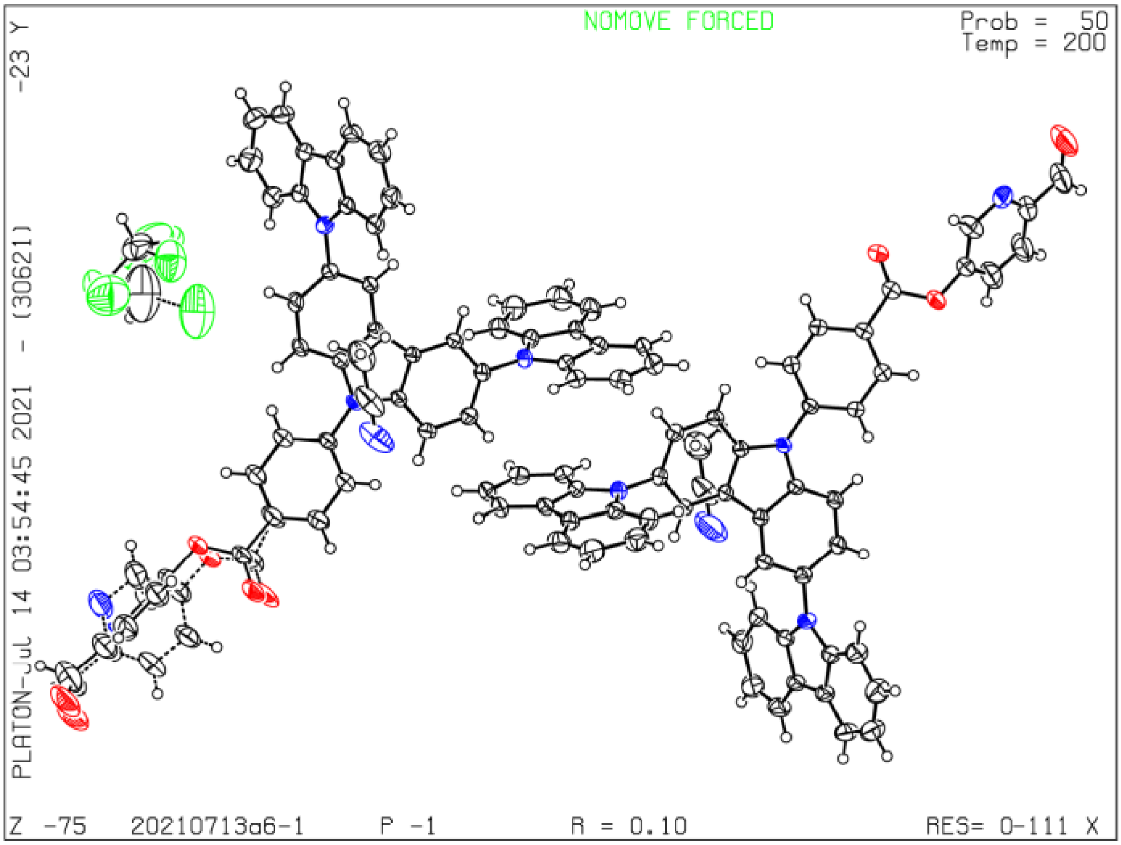

Figure S17. Crystal structure of ligand **L-A**.

7 Generation of the Molecular Model of Cage-1

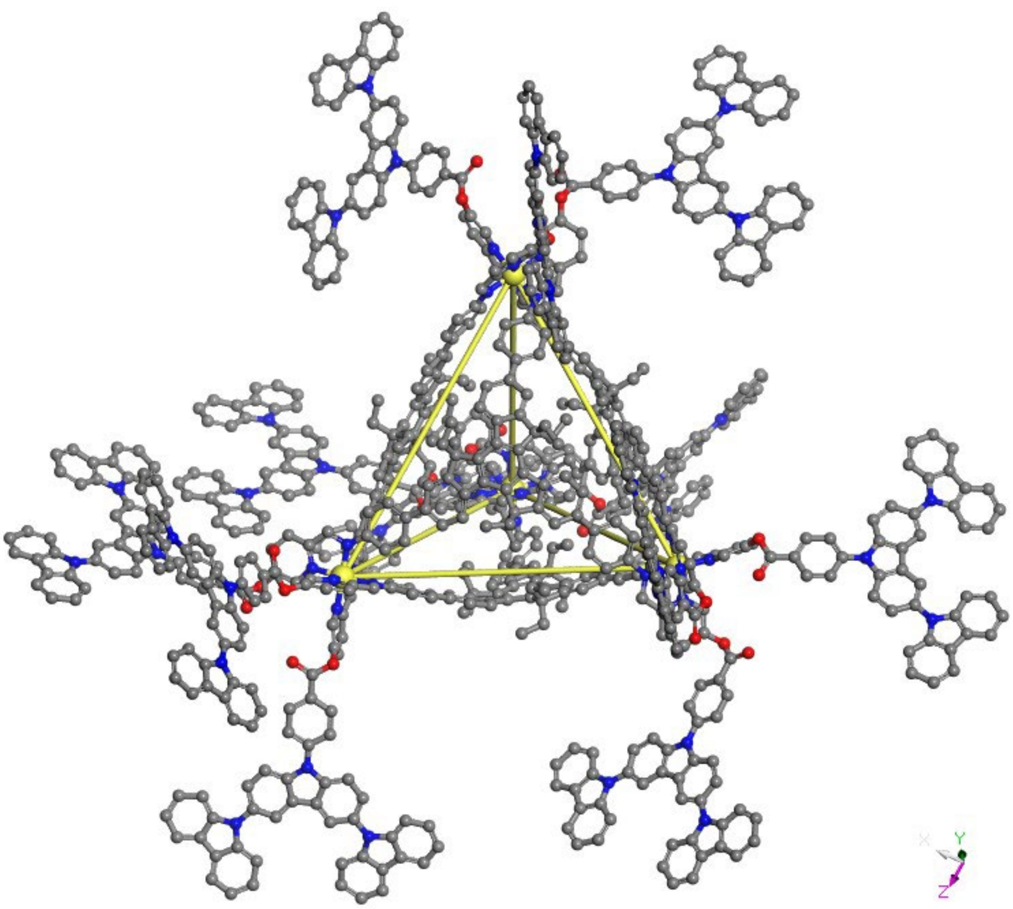

Figure S18. Energy-minimized molecular of **Cage-1** (Zn, yellow; N, blue; O, red; C, gray). Hydrogens and counteranions are omitted for clarity. **Cage-1** was measured by Materials Studio software and the structure of the simulated molecule was optimized by the Forcite module in Materials Studio software, as well as the Cartesian coordinate was marked in the lower right corner of the figure.

8 Characterization of amorphous solid Cage-1

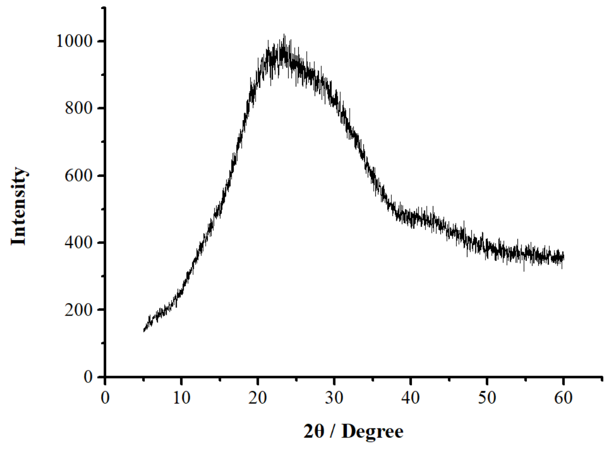

Figure S19. Experimental PXRD pattern of solid **Cage-1**.

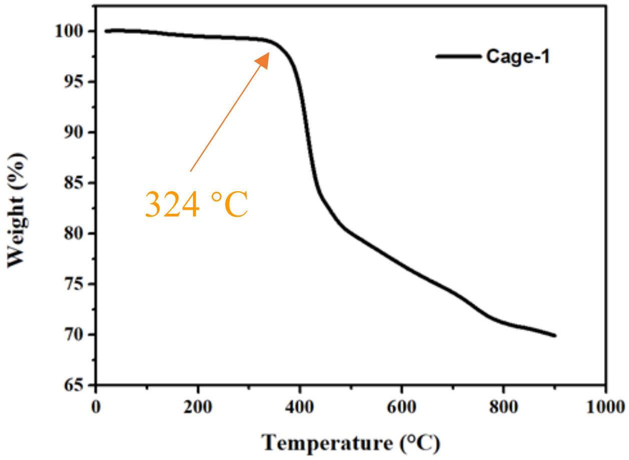

Figure S20. TGA curves of **Cage-1**. TGA was conducted at a ramp rate of 10 °C/min up to 900 °C under a nitrogen flow.

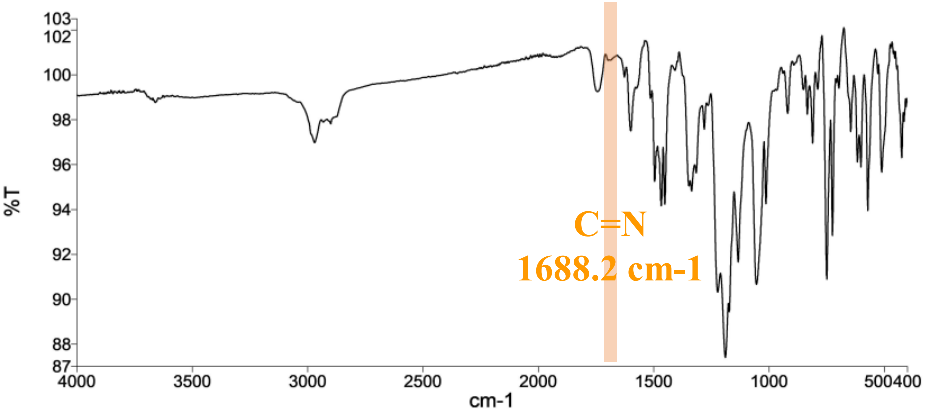

Figure S21. Fourier transform infrared spectroscopy of solid **Cage-1**.

9 Fourier transform infrared spectroscopy of solid ligands L-A and L-B

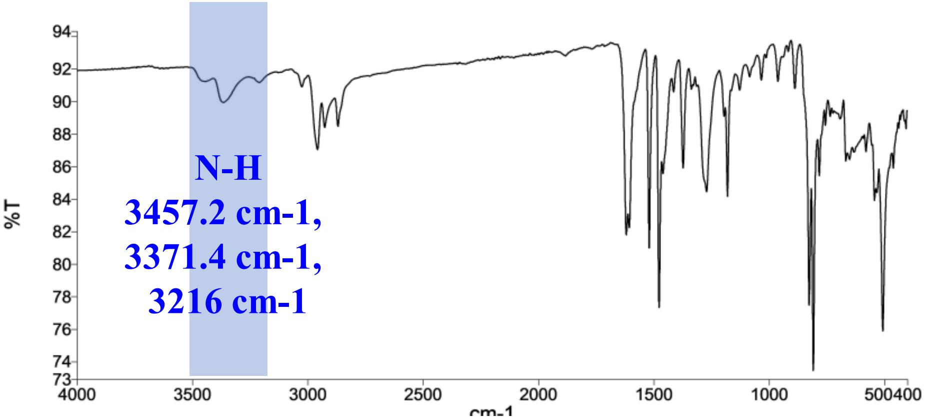

Figure S22. Fourier transform infrared spectroscopy of solid **L-B**.

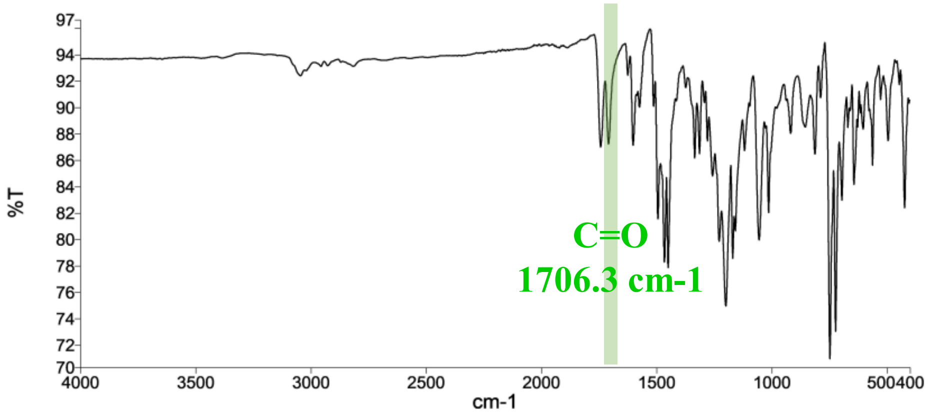

Figure S23. Fourier transform infrared spectroscopy of solid **L-A**.

10 DOSY spectra

In Stokes-Einstein equation,

$$D = \frac{k_B T}{6\pi\eta r}$$

which was applied to estimate the dynamic radius for the **Cage-1**. D is diffusion coefficient obtained from DOSY spectrum, k_B is Boltzmann constant, T is temperature, Solvent viscosity η tested to be 0.46 mPa•s, and r is the estimated dynamic radius.

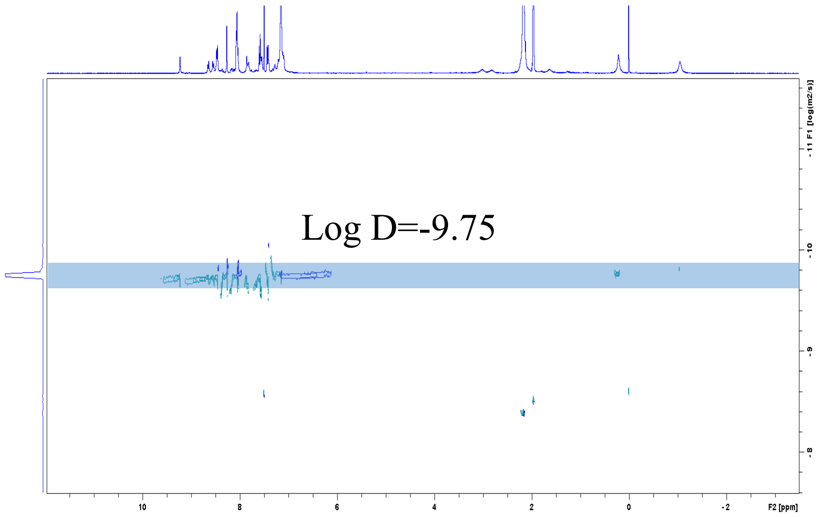

Figure S24. The DOSY spectrum of **Cage-1** (600 MHz, 298 K, $CDCl_3$: CD_3CN = 1: 1). Diffusion Constant = $1.78*10^{-10}$ m^2/s, r = 26.5 Å.

11 References

1. Yang, X.; Lu, R.; Gai, F.; Xue, P.; Zhan, Y. Rigid dendritic gelators based on oligocarbazoles. *Chem. Commun.* **2010**, *46*, 1088-1090, doi:10.1039/b918986f.

2. Qiao, S.; Wang, T.; Huang, W.; Jiang, J.-X.; Du, Z.; Shieh, F.-K.; Yang, R. Dendrimer-like conjugated microporous polymers. *Polym. Chem.* **2016**, *7*, 1281-1289, doi:10.1039/C5PY01767J.

3. Wang, X.; Wang, Y.; Yang, H.; Fang, H.; Chen, R.; Sun, Y.; Zheng, N.; Tan, K.; Lu, X.; Tian, Z., et al. Assembled molecular face-rotating polyhedra to transfer chirality from two to three dimensions. *Nat. Commun.* **2016**, *7*, 12469, doi:10.1038/ncomms12469.
